# A Method of Calculating the Kamlet–Abboud–Taft Solvatochromic Parameters Using COSMO-RS

**DOI:** 10.3390/molecules24122209

**Published:** 2019-06-13

**Authors:** James Sherwood, Joe Granelli, Con R. McElroy, James H. Clark

**Affiliations:** Green Chemistry Centre of Excellence, Department of Chemistry, University of York, Heslington, North Yorkshire YO10 5DD, UK; joegranelli32@gmail.com (J.G.); rob.mcelroy@york.ac.uk (C.R.M.)

**Keywords:** solvent effects, solvatochromism, polarity, kinetics, COSMO-RS

## Abstract

There is demand for safer and bio-based solvents, brought on by legislation and sustainability objectives. The prediction of physical properties is highly desirable to help design new molecules. Here we present an in silico approach to obtain calculated Kamlet–Abboud–Taft solvatochromic parameters using virtual experiments. The tautomerisation equilibrium of methyl acetoacetate and dimedone was calculated in different solvents with COSMO-RS theory and converted into estimates of solvent dipolarity and hydrogen bond accepting ability, respectively. Hydrogen bond donating ability was calculated as a function of the electron deficient surface area on protic solvents. These polarity descriptors correlate with rate constants and equilibria, and so ability of calculated Kamlet–Abboud–Taft solvatochromic parameters to recreate experimental free energy relationships was tested with sixteen case studies taken from the literature. The accuracy of the calculated parameters was also satisfactory for solvent selection, as demonstrated with a 1,4-addition reaction and a multicomponent heterocycle synthesis.

## 1. Introduction

The rate of a reaction [1,2], and product selectivity [3,4,5], can be favourably tuned by the astute application of the most appropriate solvent. Unlike a catalyst, a solvent also modifies equilibrium positions [6,7]. Furthermore, the solubility of substances is crucial, be it for reaction, formulation, extraction, precipitation, or liquid chromatography. Following decades of research into catalyst optimisation [8,9], solvent selection and even the design of bespoke solvents for greater reaction performance has only recently reached prominence [10,11,12,13].

Novel solvents are being commercialised and promoted in response to new regulatory restrictions on conventional solvents [14,15]. To accelerate the discovery of safer alternative solvents, reliable estimations of application performance are needed so that solvent design can be conducted in a logical way instead of arriving at acceptable replacements by trial and error. Simple and computationally fast group contribution methods are available to predict the physical properties of solvents (boiling point, density, viscosity, etc.). None of these properties reliably correlate with reaction kinetics, thermodynamics, or product yields, which ultimately determines the suitability of a solvent (assuming it is safe to use).

**Figure 1:**
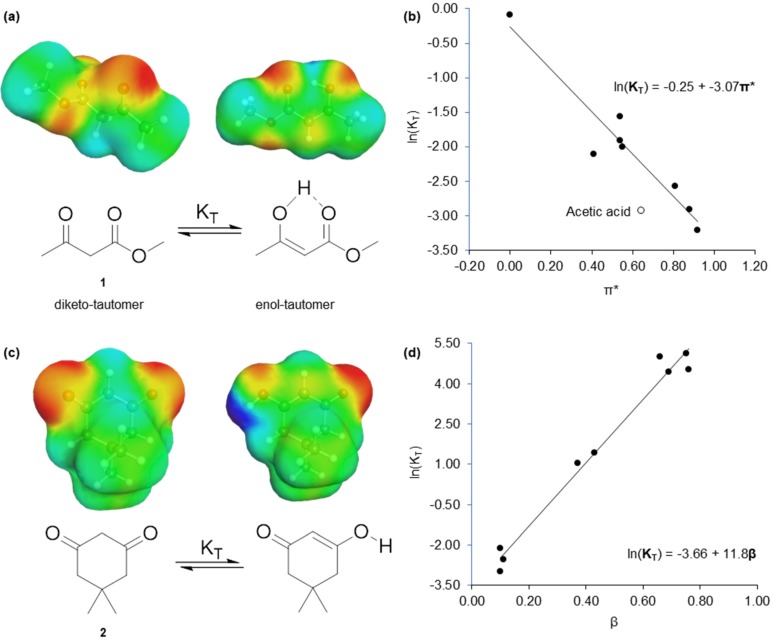
The solvent effect on the tautomerisation of acyclic and cyclic 1,3-dicarbonyl compounds. (**a**) The tautomerisation of **1**, with σ-surfaces superimposed over the lowest energy conformation of both tautomers; (**b**) The tautomerisation of **1** is inversely proportional to π* due to the smaller dipole moment of the enol-tautomer; (**c**) The tautomerisation of **2**; (**d**) The free energy relationship between the tautomerisation of **2** and β showing stabilisation of the enol-tautomer is provided by hydrogen bond accepting solvents.

Here, we report a computationally inexpensive method of predicting the Kamlet–Abboud–Taft (KAT) solvatochromic parameters of solvents [16]. The KAT parameters represent dipolarity (π*) [17], hydrogen bond accepting ability (β) [18], and hydrogen bond donating ability (α) [19]. All three are traditionally obtained from the normalised UV spectra of solvatochromic dyes. The KAT parameters correlate linearly with the logarithmic function of reaction rates and equilibria. For example, the tautomerisation of methyl acetoacetate (**1**) is a function of π*, and the tautomerisation of dimedone (**2**) is proportional to β [20] (Figure 1). To obtain calculated KAT parameters, the commercial software COSMOtherm was used to create a description of the surface charges on solvents (σ-surface) [21]. Utilising polarisation charge densities from the COSMO solvation model prior to statistical thermodynamic calculations (COSMO-RS) [22,23], an accurate representation of the type and strength of molecular interactions a solvent is capable of is provided. This is the origin of solvent polarity on a molecular scale. The aforementioned tautomerisation reactions were recreated in silico in the solvents modelled with COSMOtherm, and the calculated equilibrium constants equated to the responsible KAT parameter (π* or β) by means of a virtual free energy relationship.

We could not find a suitable molecular equilibrium that is dictated only by α. Instead, we modified the previous work of Palomar et al. that interpreted solvent polarity directly from molecular surface charges (provided in COSMOtherm as a histogram known as the σ-profile) [24]. By isolating the portion of a molecule that is capable of accepting electrons, it is possible to derive its α value. The π* and β parameters were not able to be calculated in this manner.

COSMO-RS theory has previously been used to deduce the Abraham parameters [25], and the solvatochromic response of Reichardt’s dye [24], but neither are as versatile as the KAT parameters in predicting so many types of chemical phenomena [16]. Diorazio et al. predicted KAT parameters from density functional calculations using Gaussian 09 [26]. With a similar approach Waghorne et al. predicted values of β correlating to experiment with a Pearson correlation coefficient (r) of 0.92 (after removing bases from the dataset) [27]. Our new predictive methodology serves the same purpose as the prior art by permitting the rationalisation of solvent effects, as validated in sixteen examples from the literature. Furthermore, we have conducted two new case studies for the purpose of finding a superior solvent, which was then demonstrated experimentally. With the greater predictive accuracy of our method, it was also possible to design a bespoke solvent to improve the synthesis of a substituted tetrahydropyridine compound.

## 2. Results and Discussion

### 2.1. Virtual Isomerisation Experiments

**Figure 2:**
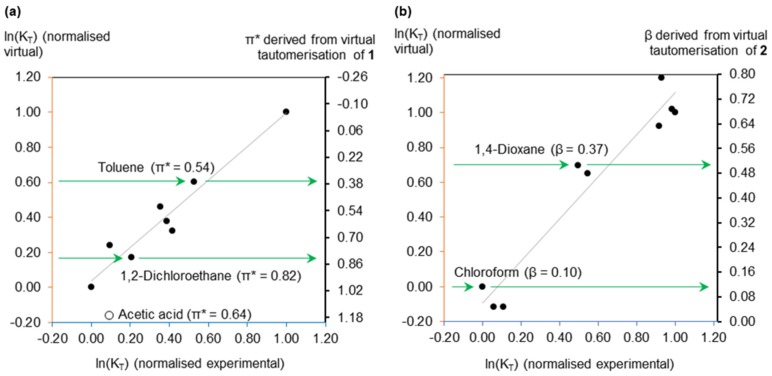
Model training datasets to establish the relationship between experimental and virtual equilibrium constants. (**a**) The π* calculation, annotated with the most accurate prediction (1,2-dichloroethane) and the least accurate (toluene). Acetic acid deviated from the trend; (**b**) The β calculation, annotated with the most accurate prediction (chloroform) and the least accurate (1,4-dioxane).

Experimental data for nine solvents were used to validate the accuracy of the calculated equilibrium constants for the tautomerisation of **1** and **2**. Linear relationships were achieved, despite the overestimation of ln(K_T_) values. This systematic error is shared with other computational methods of predicting rate constants [28]. This being the case, it is convenient to normalise the equilibrium constants to assist data visualisation and interpretation. The proportionality achieved between experimental and calculated equilibrium constants (shown in Figure 2) means the latter also correlate with π* or β. Figure 2 is also annotated with how calculated π* or β values are obtained, whereby a normalised calculated equilibrium constant corresponds to solvent polarity via the virtual free energy relationship equation.

A noteworthy observation was the in silico recreation of the experimental deviation of acidic solvents from the free energy relationship describing the tautomerisation of **1** (Figure 2a). A greater proportion of the diketo-tautomer exists in an acetic acid solution than anticipated from just the dipolarity (π*) of the solvent. This is due to the protonation of **1**, increasing the stabilisation of the diketo-tautomer compared to the enol [20]. This behaviour was validated by an additional virtual experiment in propionic acid (see Supplementary Material).

### 2.2. Dataset of Calculated Kamlet–Abboud–Taft Solvatochromic Parameters

**Figure 3:**
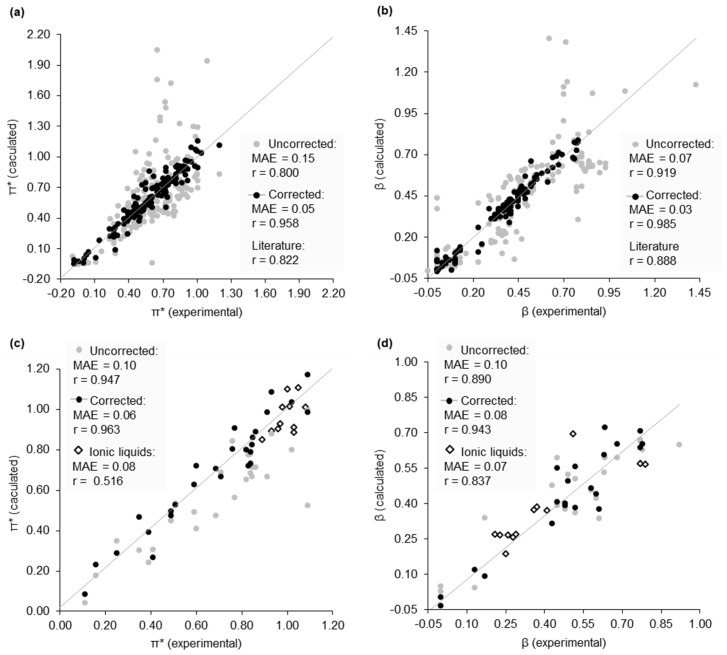
Calculated Kamlet–Abboud–Taft (KAT) parameters correlated to experimental data, accompanied by an error analysis and reference to literature methods [26]. (**a**) π* calculations, primary dataset; (**b**) β calculations, primary dataset; (**c**) π* calculations, secondary dataset, and ionic liquids; (**d**) β calculations, secondary dataset and ionic liquids.

A dataset of 175 solvents was taken from the work of Marcus [29]. This is the most complete collection of KAT parameters obtained under the same experimental conditions. Estimations of π* (Figure 3a) and β (Figure 3b) were derived from normalised virtual ln(K_T_) values using the relationship established by the training datasets (Figure 2). The dipolarity of acidic solvents could not be described for the reason already provided. This includes carboxylic acids, phenols, and fluoroalcohols. The π* values of water and perfluorinated alkanes were also overestimated. The accuracy of calculated β values was satisfactory except for amines and other highly basic (β > 0.80) solvents, at which point the model was unrepresentative. In experiment, the enol:diketo ratio of **2** in ethanol (β = 0.75) is >99%. Solvents can have a considerably higher hydrogen bond accepting ability but will only minimally increase enol tautomer concentration. By contrast, the experimental proportion of **1** in its enol form only reaches ca. 50% in low polarity hydrocarbons. It might appear that an experimental limitation is again mirrored by its virtual equivalent. However, the same issue affected the separate approaches developed by Diorazio and Waghorne (discussed in the Supplementary Material) [26,27]. This indicates it is not necessarily the use of the dimedone tautomerisation that restricts the valid range of β predictions, but perhaps a more fundamental problem of acid-base behaviour interfering with hydrogen bonding models.

**Figure 4:**
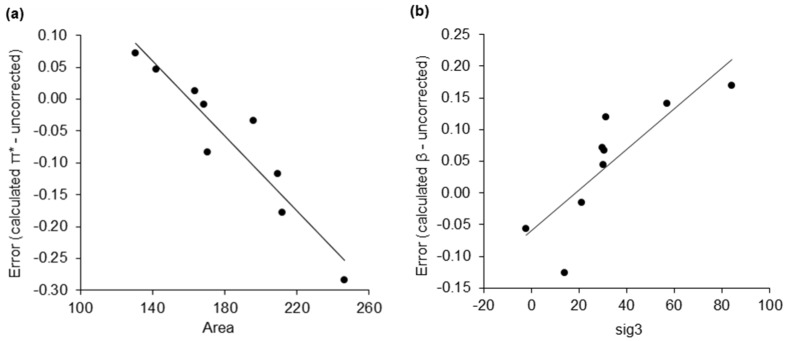
Error correction for the calculated KAT parameters of acyclic ethers as linear functions of σ-moments. (**a**) Calculated π* error correction; (**b**) Calculated β error correction.

The mean average error (MAE) of the calculated π*, β, and α values are 0.15, 0.07, and 0.06 respectively (after removing ineligible compounds). Particularly for the prediction of π* an improvement in accuracy was sought. Previously, σ-moments generated by COSMOtherm have been used to estimate the Abraham solute parameters [25]. The affinity of a solvent towards a solute (quantified as chemical potential) can be described as a function of these σ-moments (Table 1) [30]. Although these parameters alone do not directly correlate with the KAT parameters (see Supplementary Material), they can be used to correct estimations of π* and β. It was found that the π* calculation error was proportional to the molecular surface area of the solvent. Similarly, the calculation of β was improved by accounting for the asymmetry of the charge distribution on the surface of a solvent molecule. The correction is sensitive to the chemical functionality of the solvent. Using acyclic ethers as an example, the error of a calculated π* value is corrected with Equation (1) (Figure 4a), and the error of a calculated β value is corrected according to Equation (2) (Figure 4b). Figure 3a,b compare uncorrected and corrected calculated KAT parameters with experimental values for the entire Marcus dataset, with an increase in predictive accuracy compared to previously described literature methods.

π*_corrected_ = π*_uncorrected_ − (−0.0029·Area + 0.4705), (1)

β_corrected_ = β_uncorrected_ − (0.0032·sig3 − 0.0599), (2)

Corrected calculated KAT parameters are not possible to obtain for all solvents. For instance, if the number of solvents in the primary dataset with the same functionality was three or less no correction was constructed (e.g., nitroalkanes, because only data for nitrobenzene and nitromethane are available). Also, the solvent types with erroneous uncorrected calculated KAT parameters (e.g., basic solvents such as amines) could not be transformed into valid estimations. A correction was applied to the calculated α values by setting all values below 0.10 to zero, mirroring experimental practices (refer to the Supplementary Material for discussion and the full KAT parameter dataset).

The KAT parameters for a secondary dataset of 23 new solvents were then obtained. The purpose of this exercise was to verify that the correction factors are meaningful to solvents not used to define the proportionality between σ-moments and the error of calculated π* and β values. Some of these solvents were also needed for the subsequent case studies. The introduction of multifunctional compounds in this second compound set increased the prediction difficulty, but the typical error remained acceptable for uncorrected π* (Figure 3c) and β values (Figure 3d). After correction, a marginal improvement to the MAE was achieved. There is an indication that the multifunctional nature of some of these additional solvents is not addressed by the correction factors. Specifically, the correction of β values for this secondary dataset showed a bias not observed in the primary dataset, with relatively large errors remaining large (but slightly reduced on average). The failure to correct large errors may be an anomaly arising from the small dataset, or because the original representation of the solvent surface charges on the more unusual solvent molecules is inaccurate. Assuming the latter, the adequacy of the linear single variable correction factors presently used will be investigated in the future, with non-linear multiparameter models sought.

Calculated α values were more erroneous on average because some solvents in the secondary dataset are capable of intramolecular hydrogen bonds and thus appeared aprotic in their σ-profile. One example is 2-methoxyethanol, which is calculated to form a hydrogen bond resulting in a 5-membered ring structure. This conformer no longer has the ability to donate a hydrogen bond. Higher energy conformations of these solvents not featuring an intramolecular hydrogen bond could be used to obtain a realistic prediction of α (see Supplementary Material).

It was also possible to evaluate the polarity of ionic liquids. Alternative methods are available for the calculation of β and α [31], but not π*. Although we can accurately calculate π* for the first time (Figure 3c) and also β (Figure 3d), the prediction of α failed because the electron density of cations that gives rise to an equivalent interaction to hydrogen bond donation in ionic liquids differs to neutral molecular solvents and true hydrogen bonds (see Supplementary Material). The calculation of π* and β is possible for mixtures of solvents including deep eutectic mixtures (data is provided in the Supplementary Material), but it is not possible to simply combine the σ-profiles of each component to determine α. Ionic liquid and deep eutectic mixture data were not subject to corrections.

### 2.3. Application of Calculated KAT Parameters to Free Energy Relationships

The most effective use of the KAT parameters is the construction of free energy relationships, which can have two purposes. Firstly, in the tradition of physical organic chemistry, information about the mechanism of the chemical process is revealed. Secondly, extrapolation of the free energy relationship predicts the properties of the optimum solvent that will maximise the phenomenon being measured. To establish whether calculated KAT parameters are accurate enough for this purpose, sixteen case studies from the literature were found. This exercise necessitated the use of independently chosen solvents, so it was not possible to avoid solvents with the least accurately calculated KAT parameters. The variable described by each free energy relationship (e.g., ln(k), ΔG°, etc.) was correlated with both experimental and calculated KAT parameters. Typically, the weakest correlations were obtained with uncorrected calculated KAT parameters. After correction, the calculated KAT parameters generally approached the accuracy of the corresponding empirical free energy relationship based on experimental KAT parameters. All the free energy relationships are explained in the Supplementary Material.

**Scheme 1:**
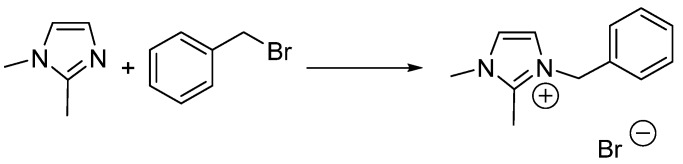
The Menschutkin reaction between 1,2-dimethylimidazole and benzyl bromide.

To illustrate with one of the sixteen case studies, the kinetics of a Menschutkin reaction between 1,2-dimethylimidazole and benzyl bromide (conducted by Skrzypczak and Neta, Scheme 1) [32], is accelerated by solvents with high π* values and decelerated by hydrogen bond donating solvents as represented by the empirical free energy relationship in Equation (3).

ln(k) = −11.27 + −2.62α + 5.94π*,(3)

**Figure 5:**
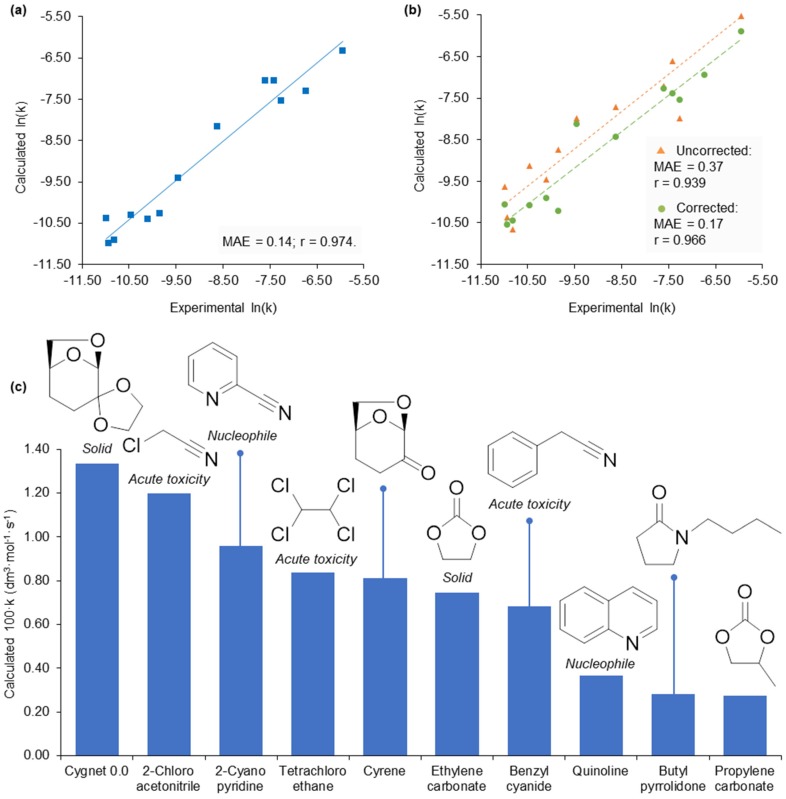
A demonstration of solvent selection for a Menschutkin reaction. (**a**) Comparison of experimental ln(k) and calculated ln(k) values (based on Equation (3) solved with experimental KAT parameters). (**b**) Substitution with calculated KAT parameters for the prediction of ln(k) and compared to experimental data. (**c**) The ten solvents with the highest predicted rate constants, annotated with practical issues and molecular schemes. Cygnet 0.0 is spiro-6,8-dioxabicyclo[3.2.1]octane-4,2′-[1,3]dioxolane [35], and Cyrene™ is dihydrolevoglucosenone [33].

They found propylene carbonate provided increased rates of reaction over the conventional solvent acetonitrile. The free energy relationship was computed using experimental (Figure 5a) and calculated (Figure 5b) KAT parameters with Equation (3) to obtain calculated ln(k) values. The corrected calculated KAT parameters, in particular, were able to replicate the solvent effect quantified by the empirical free energy relationship. Screening the dataset of calculated KAT parameters now at our disposal, the potentially most beneficial reaction solvents were found by solving the free energy relationship. Out of 198 candidates (175 from the Marcus dataset and the 23 additional solvents), propylene carbonate provided the tenth largest predicted ln(k) and acetonitrile the 50th. Examining the top ten solvent candidates and removing those that are either solid at the reaction temperature of 21 °C, nucleophilic and thus reactive, or severely toxic, only dihydrolevoglucosenone (Cyrene™) and N-butyl pyrrolidone remain alongside propylene carbonate (Figure 5c). This output is vindicated by existing studies of the Menschutkin reaction (albeit under different reaction conditions), where Cyrene™ [33], and *N*-butyl pyrrolidone [34], both considered greener alternatives to conventional dipolar aprotic solvents [14], provided greater rates of reaction than acetonitrile (82% and 31% increase to rate constant magnitude under their respective reaction conditions).

This success of this model can be compared to when density functional calculations have been employed to predict the rate of the Menschutkin reaction and also identify an optimum solvent [28]. After normalising predicted rate constants using experimental data (a necessary step in common with our approach), a 30% increase to the rate constant was predicted in nitromethane compared to acetonitrile (40% observed experimentally). This result is consistent with Abraham’s earlier experimental work on the Menschutkin reaction [36]. By limiting optimisation opportunities to conventional solvents, substitution is effective from a reaction performance perspective but not necessarily desirable with respect to human health. Nitromethane is a suspected carcinogen and may exhibit reproductive toxicity. By contrast, Cyrene™ and *N*-butyl pyrrolidone have acceptable toxicity hazards and accordingly are licensed for multi-tonne production (in accordance with EU REACH regulation).

### 2.4. 2-Methyltetrahydrofuran Identified as a Rate Accelerating Solvent in Michael Addition Chemistry

**Scheme 2:**
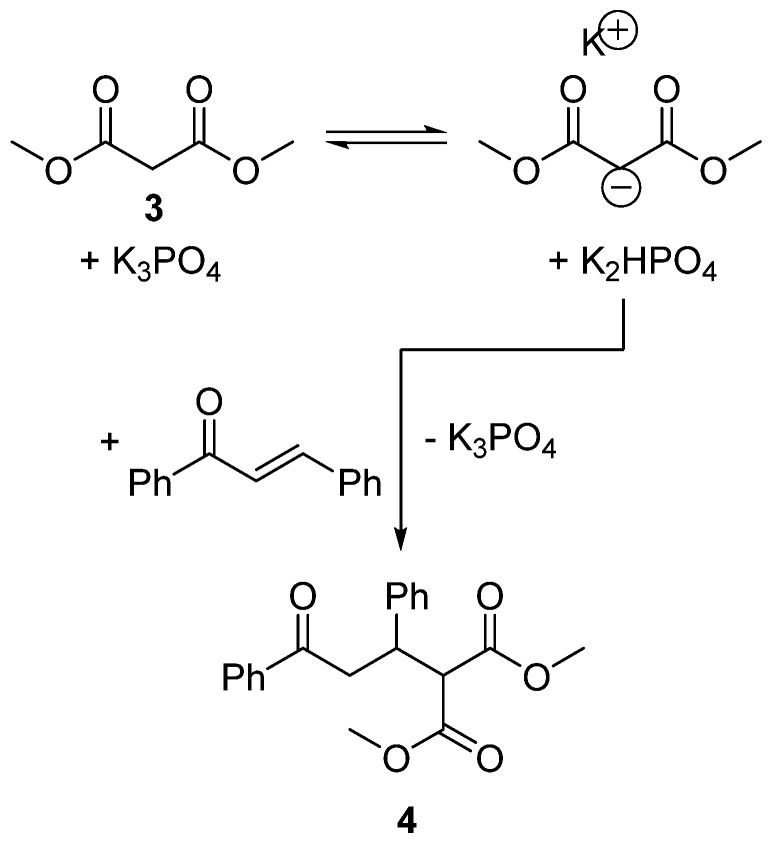
The reaction between trans-chalcone and dimethyl malonate (**3**).

The Menschutkin reaction is an uncomplicated reaction with many kinetic studies available in the literature and a strong history of empirical and computational analysis that makes solvent substitution reasonably straightforward. To provide a sterner test for this new methodology, a new experimental dataset for a reaction with previously unassessed solvent effects was created and used to identify a superior solvent. For this purpose, the kinetics of a Michael addition catalysed by potassium phosphate was measured in six solvents (Scheme 2). 1,4-Addition reactions are widely used in drug discovery and studied in the development of enantioselective catalysis [37]. It was found that the rate of the reaction favoured high β values and small solvent molar volumes (V_M_) according to Equation (4).

ln(k) = −5.16 + 5.59β + −0.0385V_M_, (4)

In this reaction, a hydrogen bond accepting solvent may interact with the conjugate acid of the catalyst to favour the deprotonation of **3** and consequently the formation of **4**. The molar volume term is indicative of a bimolecular reaction in which the cavity occupied by the reactants in solution is reduced in size upon forming a single activated complex.

**Figure 6:**
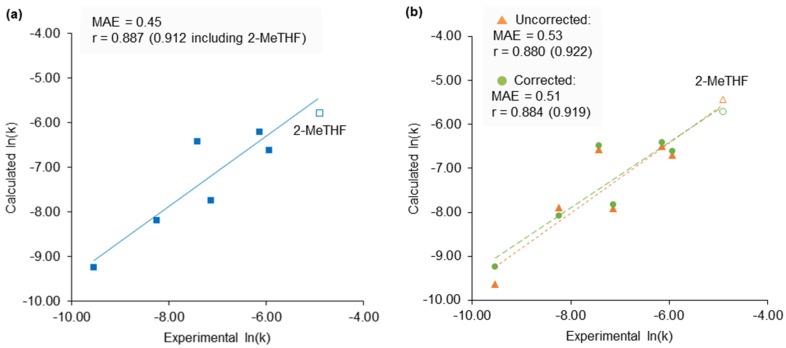
The predicted rate of a Michael addition using experimental and calculated KAT parameters. (**a**) Reaction rate constants calculated using experimental KAT parameters in Equation (4). The trendline is calculated without 2-MeTHF and extrapolated; (**b**) Calculated KAT parameters used to solve Equation (4) for the prediction of ln(k) and compared to experimental data. Trendlines are calculated without 2-MeTHF and extrapolated.

Calculated KAT parameters and predicted molar volumes obtained in COSMOtherm were used to calculate ln(k) from the free energy relationship in Equation (4), which correlated to experimental values with sufficient accuracy to screen for potential new solvents (Figure 6). To increase the rate of this reaction, the required combination of a strongly hydrogen bond accepting solvent that is also a small molecule indicated 2-methyltetrahydrofuran (2-MeTHF) as a good candidate. It is bio-based and has become a popular replacement for traditional ethers in process chemistry [38]. By extrapolating the empirical relationship in Equation (4), an accelerated rate of reaction was predicted in 2-MeTHF over the previous best solvent (dimethyl carbonate) of 131%. When using the corrected calculated β value of 2-MeTHF and its calculated molar volume, a rate enhancement of 150% is predicted. Experiment found the actual rate constant to increase by 180%.

### 2.5. Design of a Novel Solvent for the Synthesis of Tetrahydropyridines

**Scheme 3:**
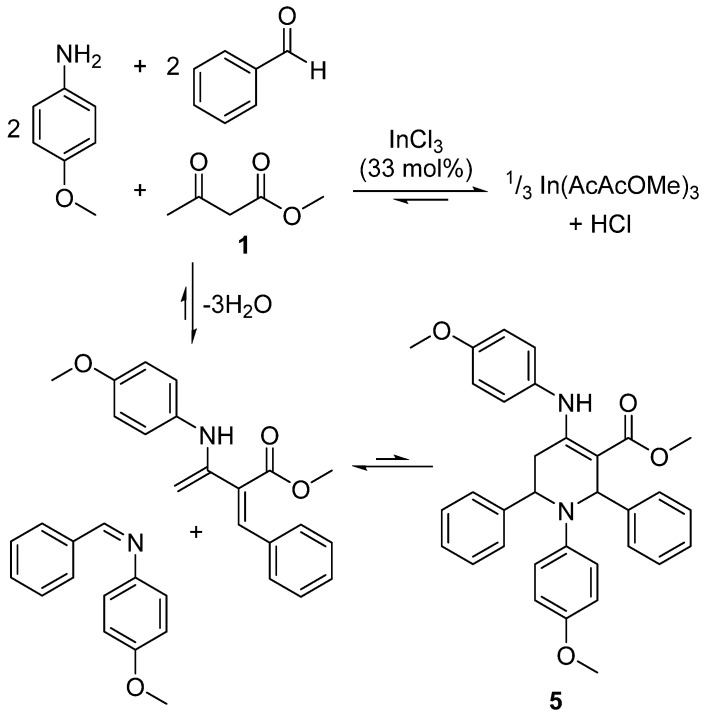
Multicomponent one-pot synthesis of tetrahydropyridine **5**.

The true value of the new method presented in this work is to identify the performance of a solvent without experimental KAT parameters before it has even been synthesised. This significantly reduces the time needed for solvent design and selection, which is critical given the regulation of popular solvents is escalating in many global territories. With this goal in mind, a reaction complicated by multiple solvent effects was chosen to demonstrate the capability of this method. The indium(III) chloride catalysed reaction between benzaldehyde, *p*-anisidine, and **1** forms a highly substituted tetrahydropyridine (**5**, Scheme 3). The product was isolated by filtration and then recrystallised. Previous reports of this reaction favour acetonitrile and methanol as yield maximising solvents [39,40].

**Figure 7:**
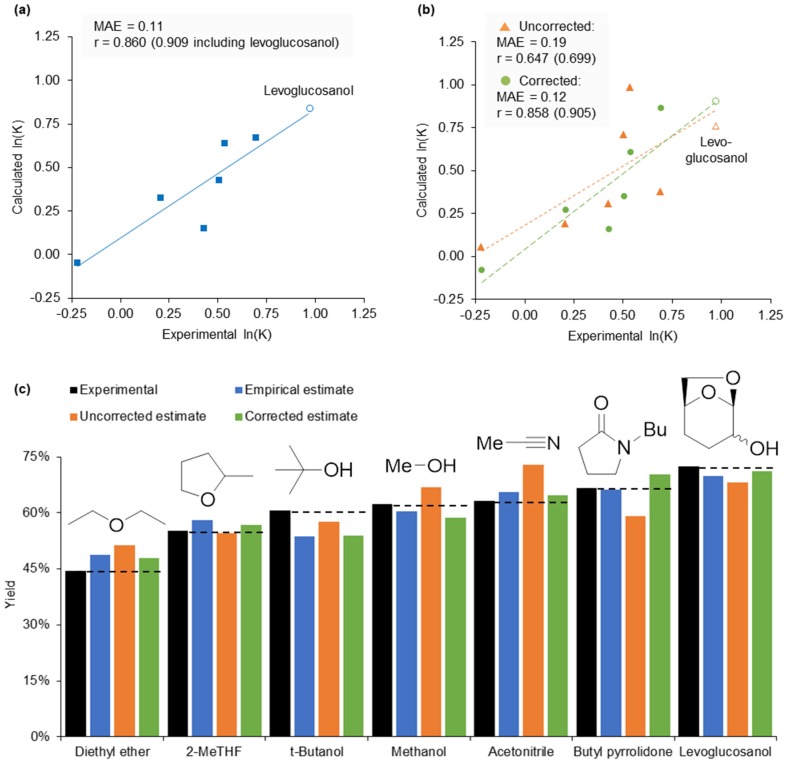
Solvent optimisation for a multicomponent reaction. (**a**) Calculation of ln(K’) from the free energy relationship in Equation (6) using experimental KAT parameters, and compared to experimental data. The trendline is calculated without levoglucosanol and extrapolated; (**b**) Calculation of ln(K’) using calculated KAT parameters. Trendlines are calculated without levoglucosanol and extrapolated; (**c**) Experimental reaction yields and predictions based on the empirical free energy relationship, and the result of the same correlation with calculated π* values.

Product yields from equilibrium-controlled reactions can be modelled as a function of solvent polarity [20]. To do so, an apparent equilibrium constant (K’) must be derived for the free energy relationship, and so a judicious choice of reaction conditions was needed to eliminate kinetic effects (provided in the Supplementary Material). Equation (5) is solved by dividing the moles of **5** (m) isolated at time *t* (i.e., the conclusion of the reaction) by the molar amount of **1** (n) not incorporated into the product (inferred by subtracting the molar yield from the initial quantity of yield-limiting **1**). After fitting ln(K’) to the empirical KAT parameters in the same manner as for other free energy relationships, experiment showed the yield increased with greater solvent dipolarity (Equation (6) and Figure 7a).

K’ = m_t_/(n_0_ − m_t_), (5)

ln(K’) = −0.44 + 1.44π*,(6)

For a successful reaction the diketo-tautomer of **1** is required to react with *p*-anisidine before a Knoevenagel condensation and the final cycloaddition (Scheme 3) [41]. Therefore, polar solvents that increase diketo-tautomer concentrations (Figure 1b) provide higher yields (Figure 7c). The chelation of indium by the enol-tautomer can be considered as a competing and stoichiometric reaction given the high catalyst loading. Equation (6) implies classical dipolar aprotic solvents such as dimethyl sulphoxide (π* = 1.00) will provide the greatest reaction productivity. However, the product is soluble in this category of solvents, allowing the final step of the reaction to become an equilibrium that favours the intermediates (Scheme 3), otherwise avoided if the product precipitates. Furthermore, solvents featuring a carbonyl functionality will be reactive in this case study (e.g., Cyrene™, which failed to produce any product), as will strong nucleophiles.

**Figure 8:**
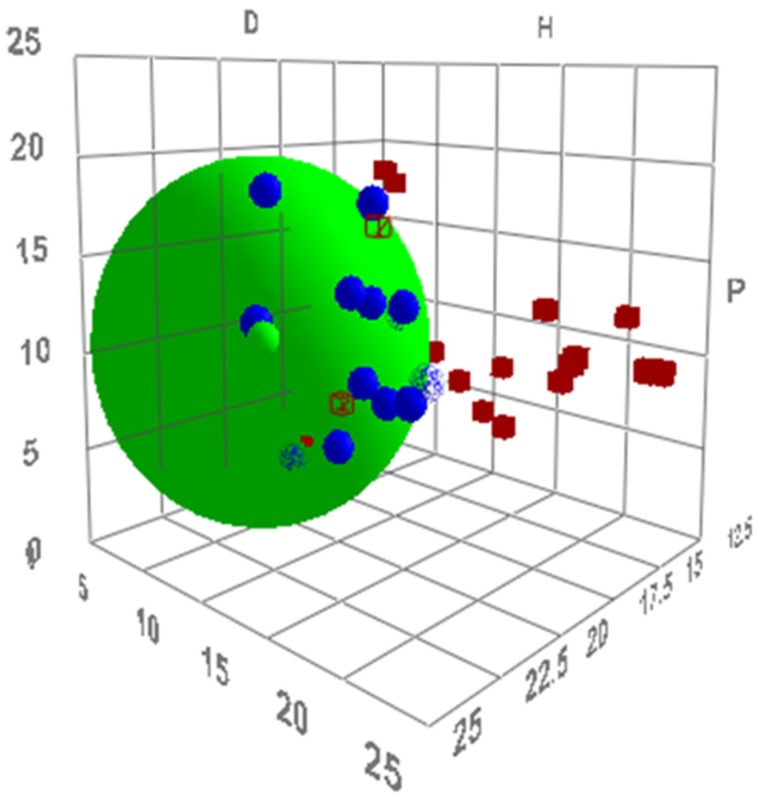
The Hansen sphere of compound **5** (green data point). Solvents are shown as blue data points and used to fit the sphere. Non-solvents are shown as red data points.

To design a stable and dipolar solvent that is unable to dissolve the product, the Hansen solubility parameters of the tetrahydropyridine were calculated from experimental solubility data using the HSPiP software (full data is provided in the Supplementary Material). The results suggested the product is not soluble in aliphatic alcohols (found outside the Hansen sphere in Figure 8). The most dipolar alcohol solvents include glycerol and other polyols, but experimental testing resulted in multiple products caused by the acetalisation and ketalisation of the reactants. The reduction of Cyrene™ created an alternative, novel solvent. The predicted π* value of levoglucosanol is 0.83, corrected to 0.93, which is greater than solvents previously shown to produce high yields. This corresponds to a predicted yield of 69% (using the uncorrected calculated π* value of levoglucosanol) or 72% (from the corrected calculated π*). The synthesis of levoglucosanol from Cyrene™ using sodium borohydride in water permitted the determination of an experimental π* value, which was 0.89. The empirical free energy relationship suggested a reaction yield of 70% would be achieved experimentally. After isolation and recrystallisation, 73% of the theoretical product yield was obtained, exceeding that observed from any of the initial solvent set and in line with predictions (Figure 7).

## 3. Materials and Methods

ArgusLab (version 4.0.1, Mark Thompson and Planaria Software LLC, 2004, Seattle, WA, USA) was used to obtain approximate atomic coordinates of compounds. The conformations of the molecules were calculated with COSMOconfX (version 4.0; COSMOlogic GmbH & Co. KG, Leverkusen, Germany, 2015). COSMOthermX (version C30_1705; COSMOlogic GmbH & Co. KG, 2017, TZVP basis set level) was used to provide molecular surface charges, σ-profiles, σ-moments, and execute the virtual experiments. Specifically, tautomerisation equilibria of **1** and **2** were calculated using the ‘Reaction’ function of COSMOtherm in the chosen solvent. In each case, the diketo-tautomer was selected as the reactant, and the enol-tautomer as the product. Equilibrium constants were calculated with the assumption of infinite dilution at 25 °C. The calculation of α is described in the Supplementary Material, as are screenshots of the different stages and outputs of the calculations. HSPiP (version 5.0.03, Abbott and Yamamoto, Ipswich, UK, 2015) was used to create the Hansen sphere.

## 4. Conclusions

A new method for the calculation of KAT parameters with diverse uses in describing chemical phenomena has been developed. Our reliance on empirical free energy relationships remains, but now it is possible to use a known relationship between a chemical phenomenon and the solvent to design and select new solvents to maximise performance prior to making them or evaluating their polarity. This process has been used to optimise the reaction rate of a Michael addition and improve the yield of a multicomponent synthesis of tetrahydropyridines. Experimental results reported in the literature have been recreated in silico to confirm the broad applicability of this approach (including the frequently studied Menschutkin reaction), which can be adopted by other practitioners without specialised expertise in computational chemistry. Some limitations remain, specifically, the inability to model acids and bases and the need for correctional factors. COSMO-RS theory would need to be modified to eliminate these restrictions. Additional experimental data would also help to refine the correction factors, especially for multifunctional compounds. A more complex but general method of error correction needs to be developed to enhance the model. As it stands, this method is an advantageous development in the context of previous attempts to estimate KAT parameters. The ability demonstrated here to accelerate the design of a new generation of solvents will be invaluable as the restriction of toxic solvents continues to force solvent substitutions across the chemical sciences.

## Figures and Tables

**Table 1:** Definitions of σ-moments.

σ-Moment	Description
Area	Molecular surface area.
sig1	Charge (zero for organic solvents).
sig2	The global electrostatic polarity of the molecule.
sig3	The asymmetry of the σ-profile, measured by skewness.
HB_don_	Hydrogen bond donor moment.
HB_acc_	Hydrogen bond acceptor moment.

## Supplementary Materials

The following are available online. Document: Full details of the calculations and an explanation of solvatochromism and the COSMOtherm software [42,43,44,45,46,47,48,49,50,51,52,53,54,55,56,57,58,59,60,61,62,63,64,65,66,67,68]. Spreadsheet: List of experimental and calculated KAT parameters, data for 16 additional case studies, and attempts to directly predict equilibrium constants in COSMOtherm.
