# Supplementary material for: A Method of Calculating the Kamlet–Abboud–Taft Solvatochromic Parameters Using COSMO-RS
Source: Molecules. 2019 Jun 13;24(12):2209. doi: 10.3390/molecules24122209 (PMC6630472; doi:10.3390/molecules24122209)
Supplement: Supplementary file 1 [file molecules-24-02209-s001.pdf]

Supplementary Material

A method of calculating the Kamlet-Abboud-Taft solvatochromic parameters using COSMO-RS

James Sherwood,* Joe Granelli, Con R. McElroy and James H. Clark*

[a]Department of Chemistry, University of York, Heslington, North Yorkshire, UK.

*Correspondence: james.sherwood@york.ac.uk; james.clark@york.ac.uk.

Contents

Section 1. Solvatochromism and solvent effects
Section 2. Calculations
Section 3. Data
Section 4. Literature case studies
Section 5. Other methods
Section 6. Michael addition
Section 7. Multi-component heterocycle synthesis

Section 1. Solvatochromism and solvent effects

Experimental determination of Kamlet-Abboud-Taft (KAT) parameters.

N,N-Dimethyl-4-nitroaniline, *N,N*-diethyl-4-nitroaniline, or 4-nitroanisole can be used to obtain π*, a measure of solvent polarity. In this work *N,N*-diethyl-4-nitroaniline was used to obtain data for 6,8-dioxabicyclo[3.2.1]octan-4-ol (levoglucosanol). Marcus used several dyes and took an average π* value.[1] The UV absorbance of these dyes changes frequency depending on the dipolarity (or polarisability for polyhalogenated solvents or aromatic solvents) of the medium it is dissolved in. The π* scale is normalised between the response of cyclohexane (363 nm, π* = 0.00) and dimethyl sulphoxide (412 nm, π* = 1.00). Therefore it is possible for solvents to have π* values outside of this scale, should they be less polar than cyclohexane or more polar than dimethyl sulphoxide. The λ_{max} of *N,N*-diethyl-4-nitroaniline in levoglucosanol is 407 nm, equating to a π* value of 0.89.

To obtain a value of hydrogen bond accepting ability (β), a second dye is required. This is the dealkylated equivalent of the first (e.g. 4-nitroaniline or 4-nitrophenol). In non-hydrogen bonding solvents, the responses of the two dyes are linearly proportional. A deviation of the UV response from this trend is observed when hydrogen bond interactions with the solvent are possible. The magnitude of the deviation is proportional to β. The scale is normalised to hexamethyl phosphoramide (originally defined as β = 1.00, but in Marcus' data set slightly higher due to the choice of dyes). The proportionality between the pair of dyes in non-hydrogen bonding solvents is not perfect, and so very weak hydrogen bonding (β < 0.10) cannot be discerned. Marcus reported low β values due to the use of several pairs of dyes and taking the average. The λ_{max} of 4-nitroaniline in levoglucosanol is 378 nm (β = 0.58).

Reichardt's dye is used to obtain α in an equivalent manner to how β is obtained (Figure S1).[2] Nitroanisole and Reichardt's dye have a proportional λ_{max} in aprotic solvents. Protic solvents deviate from this correlation, and the magnitude of deviation is the basis of the α scale. Methanol (α = 1.00) is indicated with an arrow on Figure S1. The base line (solid line) is subject to minor deviations which means accurate determination of very small α values is not possible. The dashed line in Figure S1 marks the α = 0.10 equivalence line, which is within the accuracy of the base line. It is common to assign α values below 0.1 (including negative values) as zero for this reason. An empirical equation is also available to separate the effect of solvent dipolarity from the response of Reichardt's dye to give the α value of a solvent if π* is already known.[3]

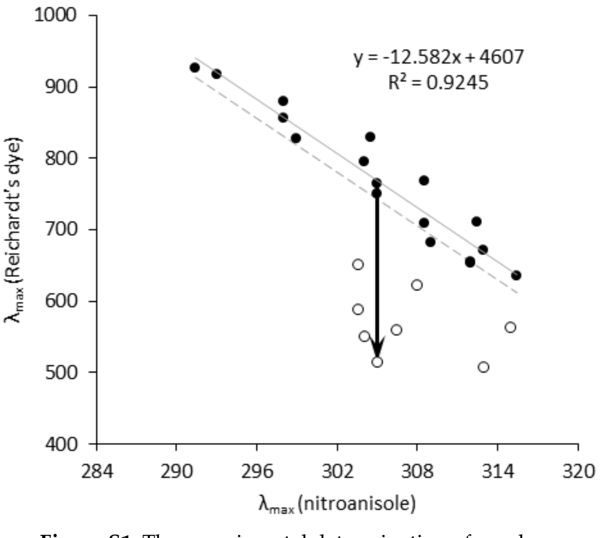

Figure S1. The experimental determination of α values.

The large dataset of Marcus reports the average KAT parameter (π^*, β, α) obtained from up to five dyes.[1] This elaborate approach is not practiced anymore. Instead, one set of dyes is used as described above.[4] This means that different KAT parameter values will be reported for the same solvent depending on the author and their methods. Using a dataset with KAT parameters from different sources introduces an inconsistency and should be avoided if possible. We required a large dataset for this work and so used Marcus' data. This means the calculated KAT parameters are predictions of the average response of different dyes. Out of necessity, when we have tested the predictive model on a secondary dataset, the prediction is of what the Marcus average value would have been and not the value we have at our disposal. This needs to be considered when applying this work and evaluating the error of the calculation. Fortunately in the majority of cases the choice of dyes does not have a dramatic impact on the values of the KAT parameters.

Tautomerisation and an explanation of the solvent effect.

The reactions chosen as the basis for solvent polarity estimations were the tautomerisation of 1,3-dicarbonyl compounds. There is a quantitative linear correlation between keto-enol tautomerisation equilibria, expressed as $\ln(K_T)$, and solvent polarity.[5] This is an example of a linear solvation energy relationship (LSER), a sub-set of free energy relationships with the following general form where XYZ is an energy dependent variable (e.g. log(k), ln(K)) and XYZ_0 is a constant (see equation 1). Other variables may be included if statistically relevant, such as molar volume to account for cavity effects.

$$XYZ = XYZ_0 + a\cdot\alpha + b\cdot\beta + s\cdot\pi^* \quad (1)$$

If the dicarbonyl compound is acyclic, such as methyl acetoacetate, enol concentration is inversely proportional to solvent dipolarity (π^*). The enol-tautomer forms an intramolecular hydrogen bond, and this species has a lower value dipole than the diketo-tautomer. Thus non-polar solvents favour higher concentrations of the enol-tautomer. If the dicarbonyl compound is cyclic (e.g. dimedone), the equilibrium position is proportional to the solvent's hydrogen bond accepting ability (β). This is because the enol-tautomer is conformationally restricted, meaning an intramolecular hydrogen bond is impossible (Scheme S1). Instead a hydrogen bond with a solvent molecule occurs. As a consequence the concentration of the enol-tautomer is higher in hydrogen bond accepting solvents.

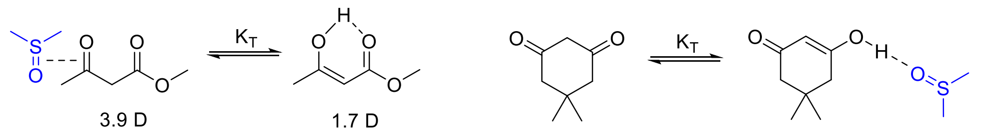

Scheme S1. The tautomerisation of methyl acetoacetate (left) and dimedone (right), with a solvent interaction shown with a molecule of dimethyl sulphoxide, a highly dipolar and hydrogen bond accepting solvent. Dipole moments of methyl acetoacetate were calculated in ArgusLab.

Software.

ArgusLab (version 4.0.1, Mark Thompson and Planaria Software LLC, 2004) was used to obtain approximate atomic coordinates of compounds. The conformations of the molecules (methyl acetoacetate, dimedone tautomers, and solvents) were calculated with COSMOconfX (version 4.0; COSMOlogic GmbH & Co. KG, 2015). COSMOthermX (version C30_1705; COSMOlogic GmbH & Co. KG, 2017, TZVP basis set level) was used to provide molecular surface charges (σ-surfaces and σ-profiles), σ-potentials, σ-moments, and execute the virtual experiments. Solvents in the default library of compounds within COSMOtherm were used where available.

Figure S2 shows representative σ-surfaces, σ-profiles, and σ-potentials. Qualitative and quantitative descriptions of these calculations can be found elsewhere.[6,7,8,9,10] To briefly summarise, the foremost output of COMSOtherm is the σ-surface, a polarity map of the surface of a molecule. Red represents electron rich regions of the molecular surface, green is neutral and blue areas are electron deficient. A σ-surface is generated for each energetically relevant conformer, and thermodynamic calculations (such as the virtual equilibria calculated in this work) consider the distribution of conformers. The σ-profile is a graphical interpretation of the σ-surface, which contains information about the electron donating and electron accepting ability of the molecule. Regions to the left of the σ-profile represent electron accepting functionalities. Its integral is the electron deficient surface area. Regions to the right of the σ-profile represent electron donating functionalities. The area under this portion of the σ-profile is the electron rich surface area. The lipophilic surface area is centred. One further analysis performed in COMSOtherm is the σ-potential. The σ-potential shows the affinity (quantified as chemical potential) of a surface segment with an external screening charge. Lipophilic solvents that do not stabilise charges have characteristic “U-shaped” curves. Conversely, the σ-potential of water and alcohol solvents are the opposite, as they are able to interact favourably with positive and negative charges. The affinity of dipolar aprotic solvents towards cations and electron accepting molecules is captured with an “S-shaped” σ-potential (Figure S2).

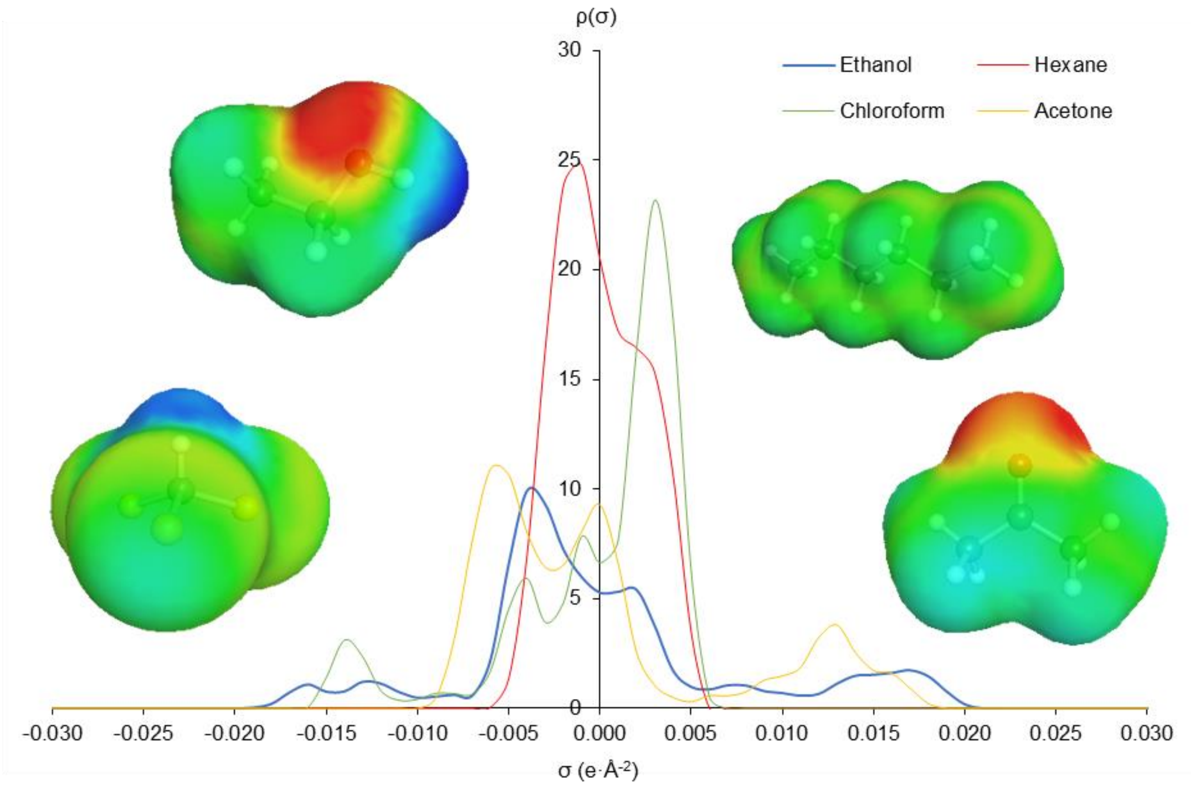

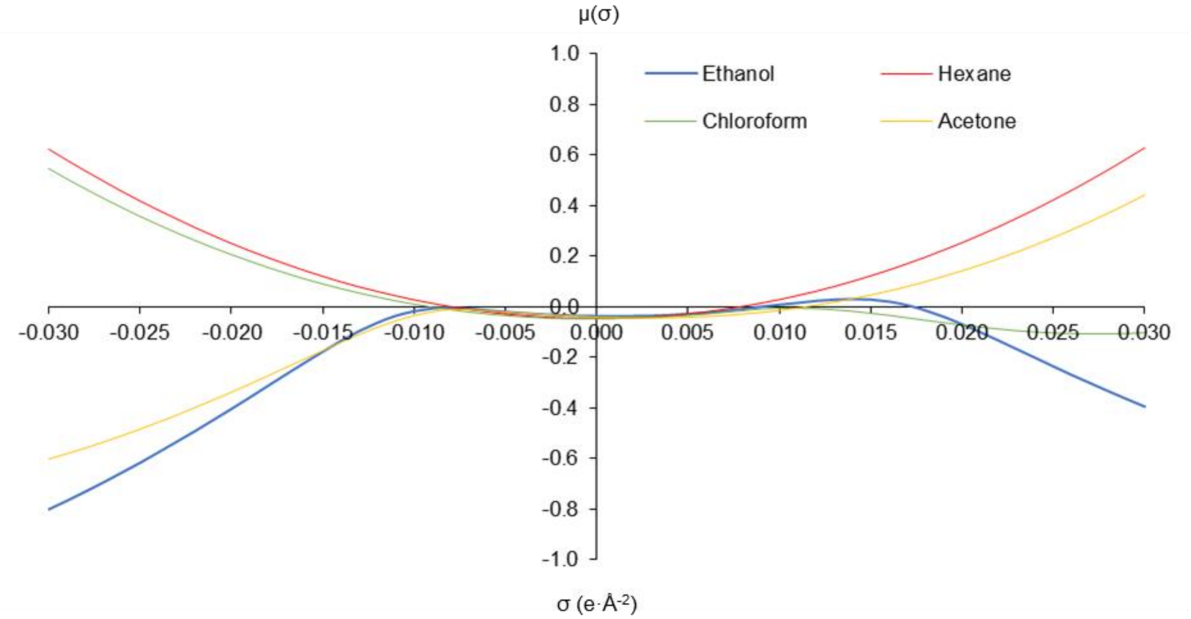

Figure S2. Top: σ-Profiles of four representative solvents, also with molecular diagrams and σ-surfaces shown. Bottom: σ-Potential of the same solvents.

Equilibria were calculated using the 'Reaction' function of COSMOtherm in the chosen solvent. Solvent densities for molar volume predictions were calculated using the 'Density' function of COSMOtherm. Solvent σ-profiles and σ-moments were accessed for each solvent and exported to Microsoft Excel. Regression analysis

to produce free energy relationships were conducted in Microsoft Excel. The P-value of variables was used to confirm statistical significance. Screenshots of the calculation of the virtual equilibrium constant for dimedone in levoglucosanol are provided in Figure S3. COSMOtherm can be run on the Microsoft Windows operating system and has a user-friendly interface that allows organic chemists and other non-computational chemists to explore solvent effects and design solvents without needing to understand the underlying theory or to write in source code.

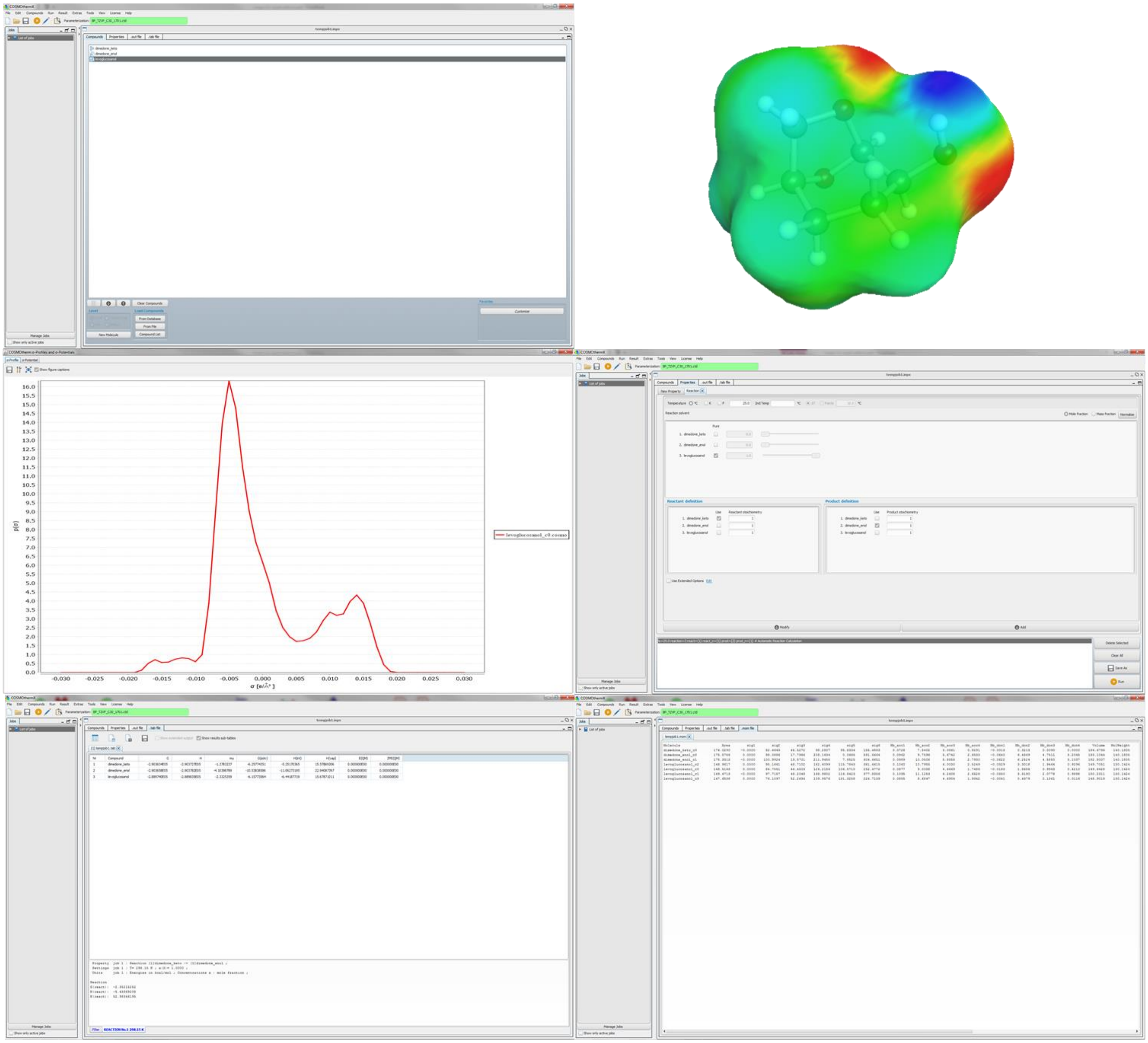

Figure S3. COSMOtherm screenshots. Top left: Compound list (dimedone tautomers and levoglucosanol). Top right: σ-Surface of levoglucosanol (lowest energy conformation, major diastereoisomer). Middle left: σ-profile of levoglucosanol (lowest energy conformation, major diastereoisomer). Middle right: 'Reaction' definition, choosing levoglucosanol as the solvent and the keto-tautomer of dimedone as the reactant and the enol as the product at 25 °C. Bottom left: 'Reaction' calculation (virtual K = 53 kcal·mol^{-1}). Bottom right: σ-Moment output screen.

For the multi-component heterocycle synthesis, the predicted solubility of the product was required to enable solvent selection. The Hansen solubility parameters of solvents were calculated using HSPiP (5th edition 5.0.03, developed by Abbott, Hansen and Yamamoto) utilising the Y-MB neural network molecular breaking technique, if not available in the HSPiP database. The Hansen solubility parameters of the solute were calculated after fitting a solubility sphere to the experimental solubility data (see Section 7).

The π* calculation.

Experimental data for the K_T of methyl acetoacetate is available for a small selection of solvents,[5] and exhibits an inverse correlation with the dipolarity of the solvent. The same trend is calculated by COSMOtherm (Table S1). Due to the difference in magnitude between experimental and virtual equilibrium constants, the former were normalised by scaling between the smallest and largest value, thereby producing a scale between zero and one. Virtual equilibrium constants were consequently adjusted by preserving the relationship to experimental values. The normalised calculated equilibrium constants, expressed as $\ln(K_T)$, were plotted against experimental π* values. The resulting linear relationship provides estimates of π* simply by using the gradient and intercept values to transform a normalised virtual $\ln(K_T)$ value of a solvent. Acidic solvents defy the correlation due to the stabilisation of the diketo-tautomer (Scheme S2).[5] Graphical analyses are provided in Figure S4.

Table S1. Experimental and calculated π* values from the training set. Mean average error (MAE) and Pearson correlation coefficient (r) are provided for calculated data (where r corresponds to the comparison to experimental data).

	ln(K)		$\ln(K)^{norm}$		π*	
Solvent	Experimental	Calculated	Experimental	Calculated	Experimental	Calculated (uncorrected)
Cyclohexane	-0.09	10.88	1.00	1.00	0.00	-0.05
t-Butanol	-2.10	9.22	0.36	0.46	0.41	0.52
Toluene	-1.56	9.67	0.53	0.60	0.54	0.36
Ethanol	-1.91	8.80	0.42	0.32	0.54	0.66
Ethyl acetate	-2.00	8.97	0.39	0.38	0.55	0.60
1,2-Dichloroethane	-2.57	8.83	0.21	0.17	0.81	0.82
DMF	-2.91	8.55	0.21	0.24	0.88	0.74
Ethylene glycol	-3.21	7.81	0.00	0.00	0.92	1.00
Propionic acid	-2.35	7.73	0.28	-0.03	0.58	1.03
Acetic acid	-2.92	7.33	0.09	-0.16	0.64	1.63
MAE		11.07		0.06		0.09
r	0.967		0.967		0.938	

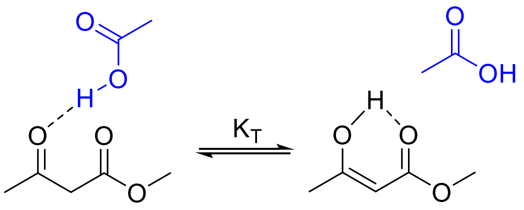

Scheme S2. The interaction that stabilises the keto-tautomer of methyl acetoacetate in acidic solvents, illustrated for a solution in acetic acid.

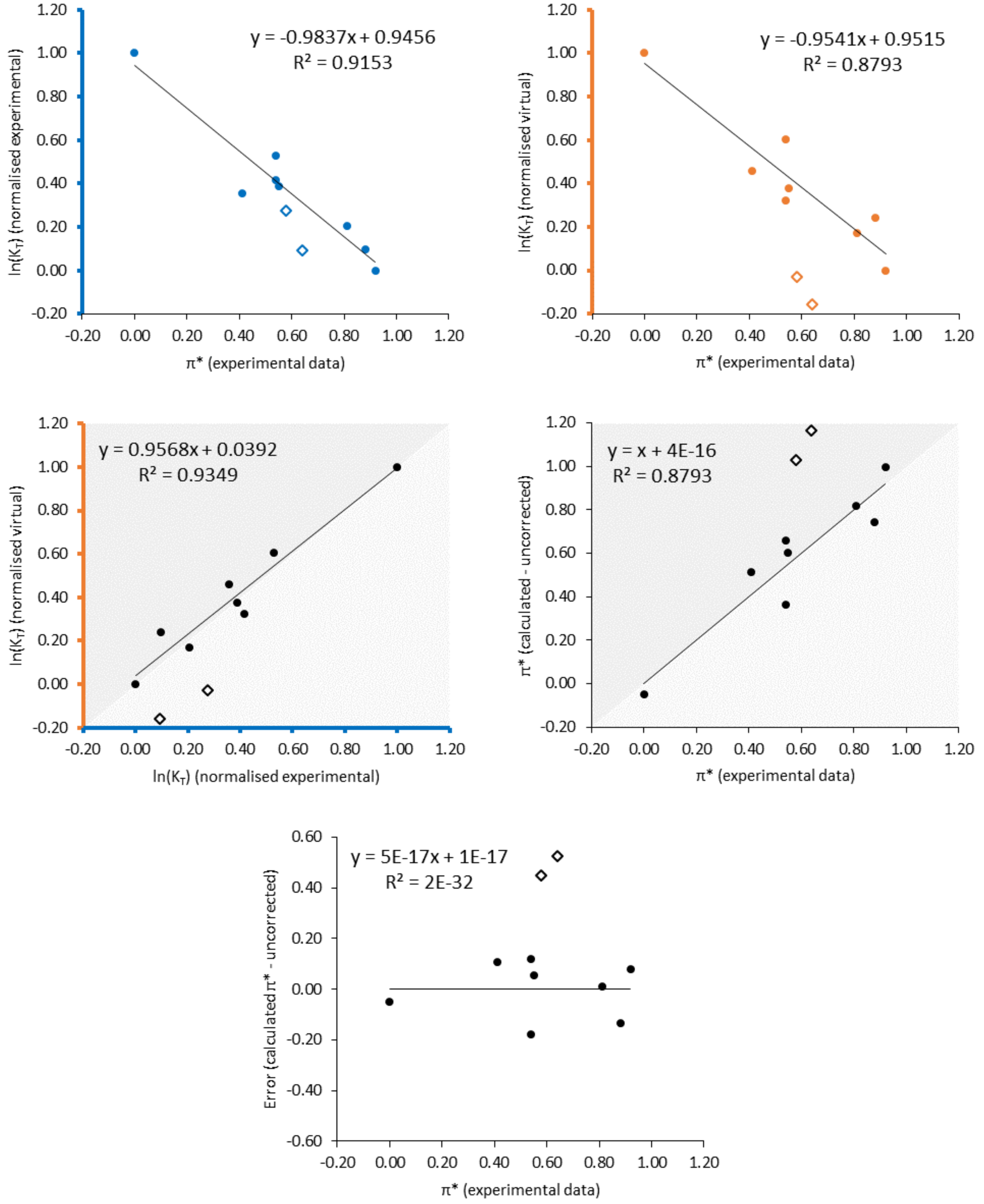

Figure S4. Top left: Empirical relationship between π^* and the tautomerisation of methyl acetoacetate. Top right: The virtual relationship between experimental π^* values and methyl acetoacetate tautomerisation calculated in COSMOtherm. Middle left: Proportionality between experimental and virtual equilibrium constants. Middle right: Experimental versus calculated π^* values. Bottom: Systematic error analysis. Acidic solvents are shown by diamond-shaped datapoints.

The β calculation.

The method of calculating β is identical to that of π* except dimedone is now the substrate (Table S2). The equilibrium is directly proportional to β. The solvent training set does not include highly basic solvents (β > 0.8). Graphical analyses are provided in Figure S5. The issue of basic solvents disobeying the predictive method was also experienced by Diorazio,[11] and Waghorne.[12] No resolution had been found. Diorazio *et al.* examined just four basic solvents, achieving one satisfactory prediction of β and three with errors exceeding 0.20.[11] Waghorne *et al.* investigated a larger number of basic solvents, but alcohols and amines were not modelled correctly and removed from subsequent analysis.[12]

Table S2. Experimental and calculated β values from the training set.

	ln(K)		$\ln(K)^{norm}$		β	
Solvent	Calculated	Experimental	Calculated	Experimental	Experimental	Calculated (uncorrected)
Benzene	-2.12	0.11	-0.18	-0.12	0.10	0.05
Chloroform	-3.00	0.00	0.57	0.00	0.10	0.11
Toluene	-2.53	0.06	-0.18	-0.12	0.11	0.05
1,4-Dioxane	1.03	0.50	5.09	0.70	0.37	0.50
Acetone	1.44	0.55	4.79	0.65	0.43	0.48
Methanol	5.00	0.98	7.18	1.02	0.66	0.69
Dimethyl formamide	4.44	0.92	6.56	0.92	0.69	0.63
Ethanol	5.13	1.00	7.05	1.00	0.75	0.67
Dimethyl sulphoxide	4.54	0.93	8.35	1.20	0.76	0.79
MAE		2.81		0.11		0.06
r	0.967		0.967		0.972	

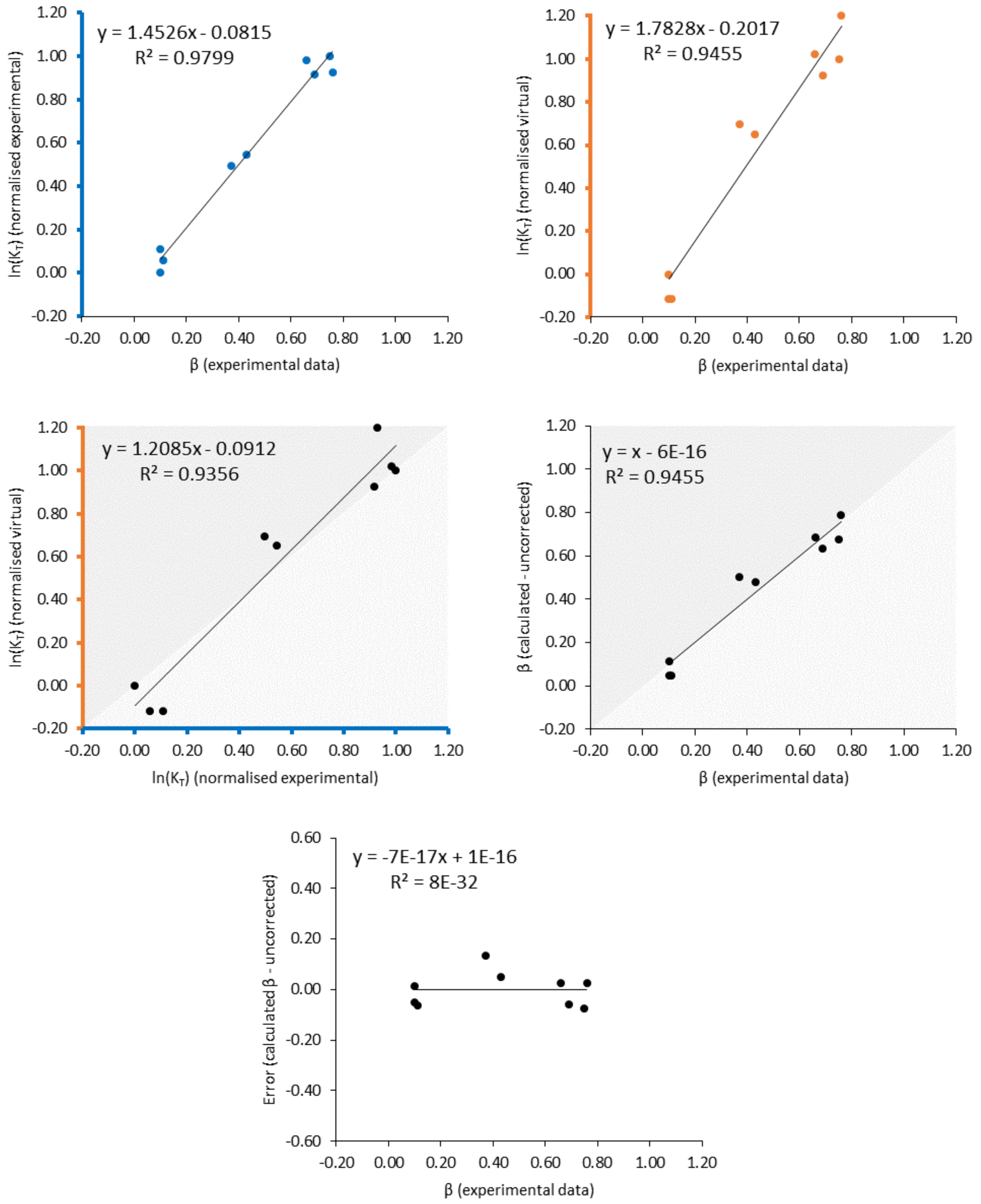

Figure S5. Top left. The empirical relationship between β and the tautomerisation equilibrium constant of dimedone. Top right: The virtual relationship between experimental β values and the tautomerisation equilibrium constant of dimedone calculated in COSMOtherm. Middle left: The proportionality between experimental and virtual equilibrium constants. Middle right: A comparison of experimental and calculated β values. Bottom: Systematic error analysis.

The α calculation.

The σ-profile of a solvent describes the electronic charge as a function of molecular surface area (Figure S2). The central region shows the surface area of a molecule that is neutral (uncharged). Negatively charged areas are further right, and electron deficient areas to the left. The σ-profile in this latter portion is zero for aprotic solvents. Alcohols and other protic solvents have some electron deficient surface area at the location of a hydrogen bond donating functionality (e.g. the hydrogen atom in an alcohol group). A linear regression fitting experimental α values to σ-profiles (for the lowest energy conformation of each of the 175 solvents in Marcus' dataset) revealed only a portion of the electron deficient surface area has a statistically relevant contribution to the model. This region is highlighted in Figure S6, which shows the σ-profile of ethanol. Equation 2 was subsequently used to calculate the α values of solvents. A training set was not used to obtain α values, unlike the calculation of π* and β.

$$\alpha = 0.03 - 67.42 \cdot \varrho(\sigma_{-0.023}) + 16.43 \cdot \varrho(\sigma_{-0.022}) - 0.01 \cdot \varrho(\sigma_{-0.021}) - 2.67 \cdot \varrho(\sigma_{-0.020}) + 2.71 \cdot \varrho(\sigma_{-0.019}) - 1.05 \cdot \varrho(\sigma_{-0.018}) + 1.05 \cdot \varrho(\sigma_{-0.017}) \qquad (2)$$

The model generally underestimates the α values of aliphatic monoalcohols, which in the context of the whole data set is balanced by an overestimation of aromatic alcohols, water, and occasionally phenols and carboxylic acids. Due to the data fitting exercise performed on the primary data set as a whole, aprotic solvents are assigned α values of 0.03 due to the constant in equation 2. Any solvent with a calculated $\alpha < 0.10$ has been corrected to zero, as is accepted practice for experimental values (Figure S1). One functionality that can give rise to a valid α value below 0.1 would be a ketone, due to tautomerisation. Solvents with C-H acidity, including some nitriles and chlorohydrocarbons are not recognised as having a measurable α value by this method. Marcus reports low α values because that data is the average value obtained from a number of different dyes, some of which will record relatively large α values. The largest calculation error is 2-chloroethanol. The hydrogen bond donating ability of 2,2,2-trifluoroethanol was also underestimated, but the predicted α value of hexafluoroisopropanol was correct to 2 decimal places (1.96), so there is not a systematic error for halogenated alcohols. The MAE of the α predictions was 0.06 ($r = 0.965$ when correlated to experimental values), reduced to 0.05 ($r = 0.966$) after the correction to aprotic solvents was applied. A literature method is accurate to $r = 0.944$.[11]

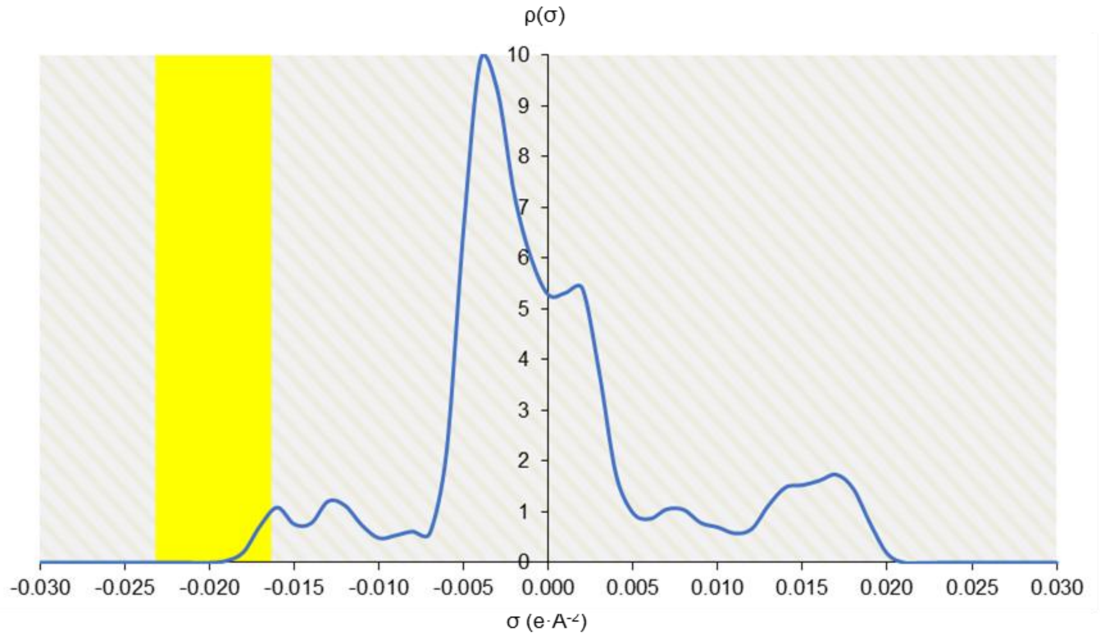

Figure S6. The σ-profile of ethanol, with yellow highlighting the portion of the histogram relevant to the calculation of α values.

This approach was adapted from the previous work of Palomar *et al*.[13] However there are some significant differences. Palomar *et al*. used the entire span of the σ-profile to deduce the response of Reichardt's dye instead of α. The UV spectrum of Reichardt's dye responds to the hydrogen bond donating ability (α) and dipolarity (π^*) of the solvent it is dissolved in, and can be used in its own right as a scale of solvent polarity.[14]

When extending the model to predict the α values of an additional 23 solvents, 2-methoxyethanol and ethyl lactate in particular resulted in poor accuracy. This was discovered to be due to intramolecular hydrogen bonds concealing the hydrogen bond donating ability of the solvent. COSMO-RS theory allows solvents to be modelled as a realistic distribution of conformers. The lowest energy conformers of 2-methoxyethanol and ethyl lactate are stabilised by intramolecular hydrogen bonds (Figure S7). This interaction is broken in the second lowest energy conformation of ethyl lactate, whereby the proton rotates out of the plane of the molecule. The proportion of electron deficient molecular surface area is now much increased. There are two conformers of 2-methoxyethanol that exhibit intramolecular hydrogen bonding, but in the third lowest energy conformation the alcohol group is directed away from the ether functionality. The difference in σ-profiles is shown with an example in Figure S7.

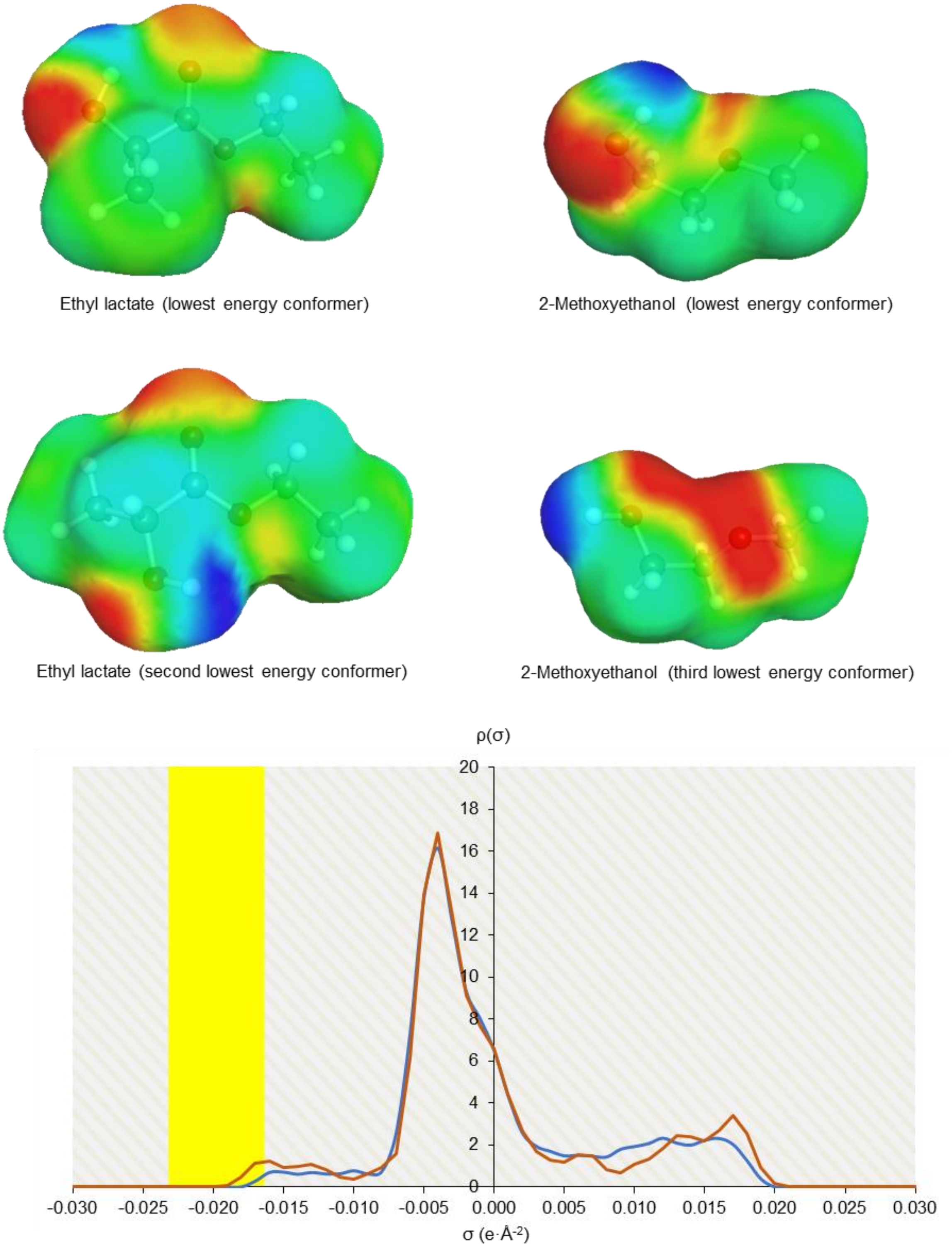

Figure S7. Top: The σ-surfaces of ethyl lactate and 2-methoxyethanol conformers. Red represents electron rich surface area, green is neutral and blue sites are electron deficient. Bottom: The σ-profile of 2-methoxyethanol intramolecular hydrogen bonding (blue) and non-hydrogen bonding (orange) conformers, with yellow highlighting the portion of the histogram relevant to the calculation of α values.

It is possible to select specific conformers for virtual experiments in COSMOtherm. The conformers of ethyl lactate and 2-methoxyethanol without intramolecular hydrogen bonding were isolated and used to provide a revised prediction of α. This resulted in an improved estimation of α (Table S3). Three further solvents in the secondary dataset also had intramolecular hydrogen bonds. In these cases, the calculation of an accurate α value was prevented because all the determined conformers were hydrogen bonding (i.e. diethylene glycol methyl ether) or very unfavourable conformations were required. High energy conformations become unrepresentative of the actual solvent composition.

Table S3. The effect of conformation on calculated α values.

Solvent	Experimental α value	Calculated α value from conformer with an intramolecular hydrogen bond	Calculated α value in the absence of an intramolecular hydrogen bond
Ethyl lactate	0.69	0.03 (corrected to 0.00)	0.51
2-Methoxyethanol	0.90	0.32	0.89
Diethylene glycol methyl ether	0.53	0.03 (corrected to 0.00)	0.06
Ethylene glycol butyl ether	0.59	0.13	0.86
Solketal	0.59	0.20	0.88

The α values of ionic liquids were not successfully calculated. Ionic liquids are typically aprotic, but interactions of the cation with electron donating molecules replicate a hydrogen bond donor interaction. As shown in Figure S8, the 1-butyl-3-methylimidazolium [bmim] cation does not have any surface area with sufficiently high and localised electron deficiency to register a non-zero α value. Instead, the positive charge is resonant, and consequently there is a large proportion of the cation surface with a weak positive charge.

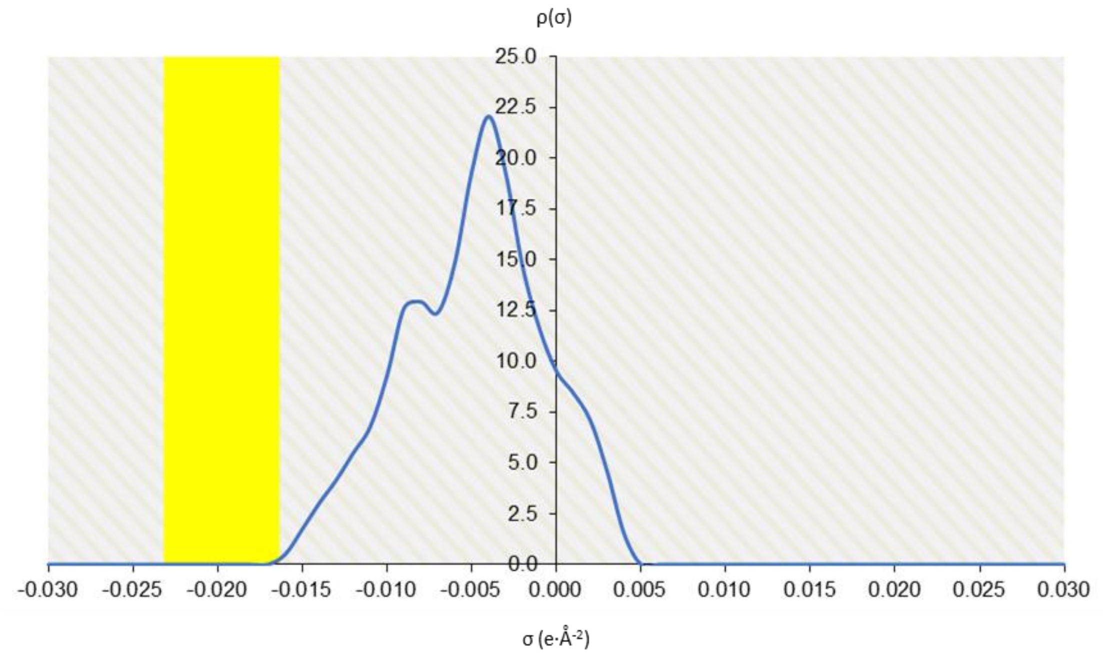

Figure S8. The σ-profile of the 1-butyl-3-methylimidazolium cation, with yellow highlighting the portion of the histogram used in the calculation of α values for molecular solvents.

Kamlet-Abboud-Taft (KAT) parameter data set.

A separate supplementary spreadsheet (Excel file) is available containing experimental KAT parameters, virtual ln(K_T) values, calculated KAT parameters and their correctional factors.

The primary solvent data set obtained from Marcus was amended in the following ways: Due to missing π^* data, styrene, furan, *n*-propyl acetate, methyl propanoate, methyl benzoate, *N*,*N*-diethyl formamide, and *N*-methyl caprolactam were removed. Eucalyptol,[15] 2-methyltetrahydrofuran,[16] ethylene carbonate,[17] and dimethyl carbonate,[18] also lacked π^* data, but alternative values were found. The contentious data for glycerol was updated to a newer version.[15] The secondary dataset,[15,16,18,19,20,21,22,23,24,25,26] and ionic liquids,[27,28,29] were collated from various sources. A systematic error analysis for uncorrected and corrected calculated π^* and β values is provided in Figure S9. A graphical analysis of corrected and uncorrected calculated α values is provided in Figure S10.

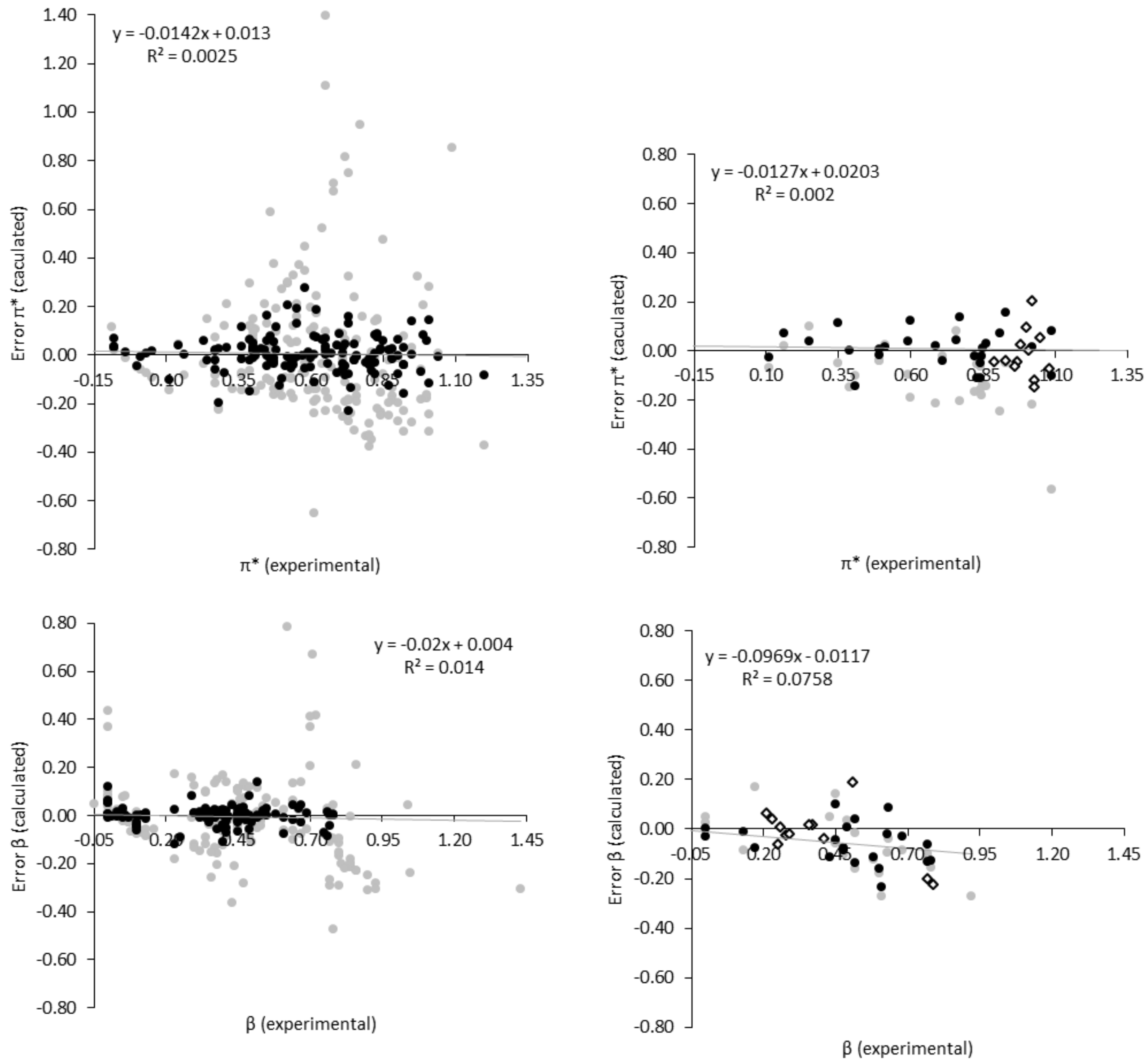

Figure S9. Systematic error checks for uncorrected (grey) and corrected (black) calculated KAT parameters. Trendline correlations (for corrected data) and coefficients of determination are given. Left: Marcus dataset. Right: Secondary dataset and ionic liquids (diamond datapoints).

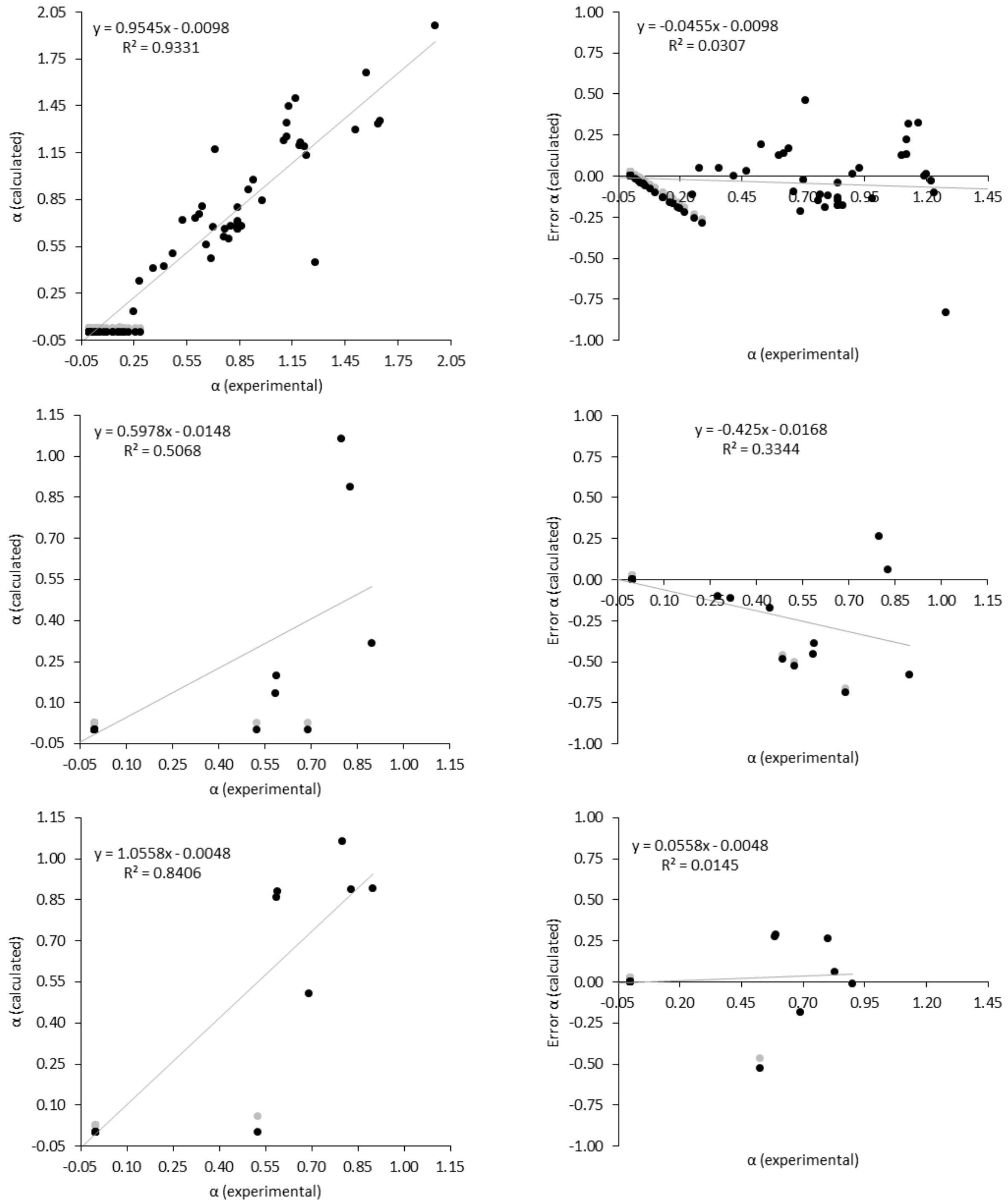

Figure S10. The comparison of experimental and calculated α (uncorrected values in grey, corrected values in black). Trendline correlations and coefficients of determination are given for corrected calculated α values. Top: Marcus dataset. C–H acids could not be predicted, and results in the linear group of errors for weak hydrogen bond donors. Centre: Secondary dataset consisting of mostly aprotic solvents and intramolecular hydrogen bonding solvents. Bottom: Secondary dataset where non-hydrogen bonding conformers have been used if applicable for the comparison of experimental and calculated α values.

Deep eutectic mixtures.

COSMOtherm allows a mixture of solvents to be chosen as the medium for a virtual reaction, and so no modification to the method is required to calculate π^* or β. The components of deep eutectic solvents interact strongly with each other, and so it was interesting to see if the polarity of representative examples could be modelled. The MAE of π^* calculations was 0.08, and for β calculations it was equal to 0.10 (reduced to 0.08 if discounting the high β values above 0.80). The data is sourced from different publications,[30,31,32] and so the consistency across the dataset is not high. The approach to determining α values is not possible to directly recreate for a mixture. The addition of two (or potentially more) σ-profiles will significantly overestimate hydrogen bond donating ability.

Table S4. A comparison of virtual equilibria with experimental data and described as a LSER.

	π^*		β	
Mixture (molar ratio)	Experimental	Calculated	Experimental	Calculated
Tetraethylammoniumchloride-Butanoic acid (1:2)	0.92	0.79	0.76	0.79
Tetraethylammoniumchloride-Hexanoic acid (1:2)	0.86	0.76	0.85	0.78
Choline chloride-Glycerol (1:3)	0.97	0.96	0.66	0.74
Choline chloride-Glycerol (1:2)	0.98	0.92	0.66	0.77
Choline chloride-Glycerol (2:3)	0.98	0.89	0.66	0.79
Choline chloride-Glycerol (1:1)	0.86	0.83	0.66	0.82
Choline chloride-Urea (1:2)	1.11	0.92	0.91	0.80
Choline chloride-Ethylene glycol (1:2)	1.02	1.03	0.91	0.77

Linear solvation energy relationships (LSERs) with experimental and calculated data.

Sixteen free energy relationships from the literature were reproduced using experimental KAT parameters, and the calculated KAT parameters (uncorrected and corrected) obtained from the virtual experiments (Scheme S3 to Scheme S16).[5,17,19,26,27,33,34,35,36,37,38] Comparing the correlations to the experimental data, XYZ (see equation 1), be it ln(k), ln(K), etc., showed a satisfactory recreation of the relationships *in-silico*. Solvents with KAT parameters that cannot be corrected were removed from the corresponding correlations. The free energy relationships were recalculated using KAT parameters preferentially from the Marcus dataset, and any coefficients with a P-value greater than 0.01 were eliminated. In case study 12 (the biocatalysed Fischer esterification) the molar volume was calculated using a predicted density from COSMOtherm. The ability of calculated KAT parameters to recreate the LSERs is assessed in the supplementary spreadsheet, where for each case study the empirical relationship is shown alongside the trends where calculated KAT parameters (uncorrected and corrected) substitute the experimental values. Case study 7 was featured in the main article.[26]

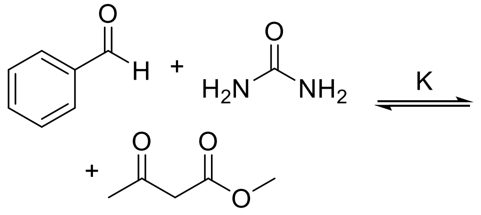

Scheme S3. Case study 1. The apparent equilibrium for the Biginelli reaction of methyl acetoacetate.[5] Catalysed by HCl.

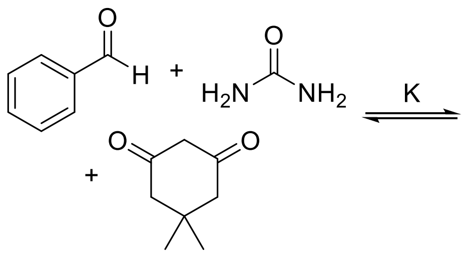

Scheme S4. Case study 2. The apparent equilibrium for the Biginelli reaction of dimedone.[5] Catalysed by HCl.

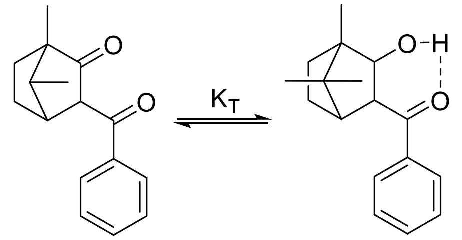

Scheme S5. Case study 3. Diketo-enol tautomerisation.[33]

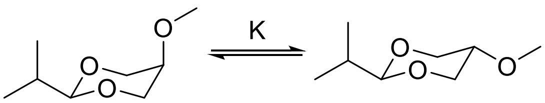

Scheme S6. Case study 4. Isomerisation.[33]

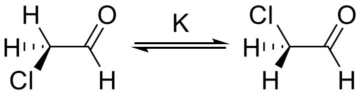

Scheme S7. Case study 5. Bond rotation.[33]

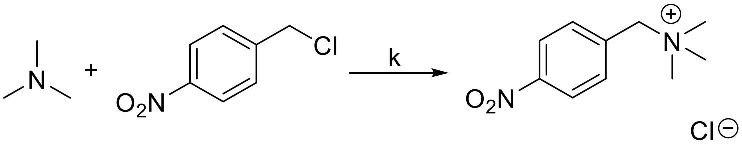

Scheme S8. Case study 6. Menschutkin *N*-alkylation of an amine.[34]

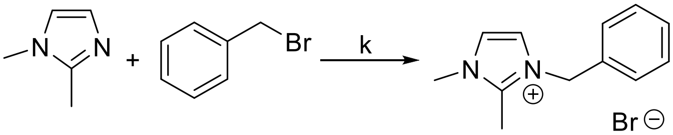

Scheme S9. Case study 7. Menschutkin *N*-alkylation of an imidazole.[26]

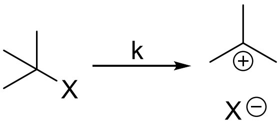

Scheme S10. Case studies 8-10. Alkylhalide solvolysis. Case study 8: X = Cl; Case study 9: X = Br; Case study 10: X = I.[35]

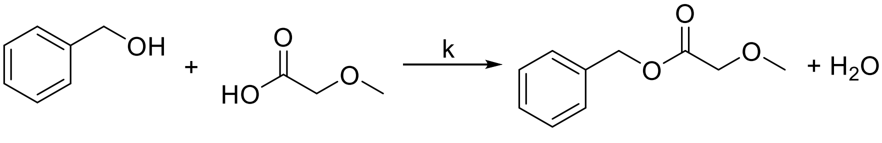

Scheme S11. Case study 11. Fischer esterification.[27] Catalysed by *p*-toluenesulphonic acid.

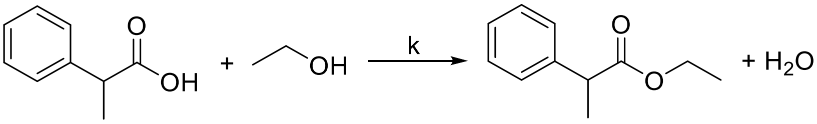

Scheme S12. Biocatalysed Fischer esterification.[36]

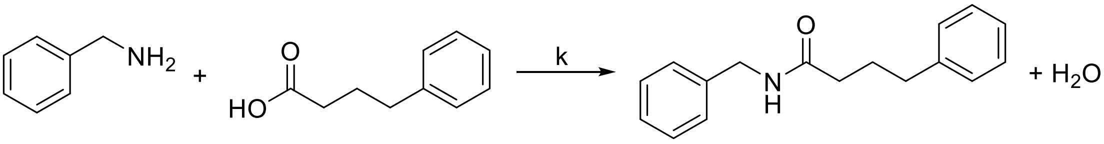

Scheme S13. The reaction rate of thermally activated amidation.[19]

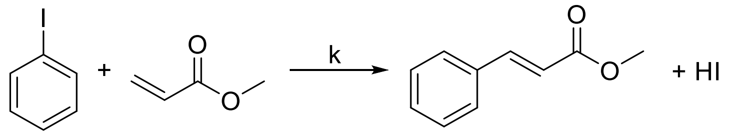

Scheme S14. Heck cross-coupling reaction.[17] Catalysed by Pd/C, with triethylamine to abstract HI.

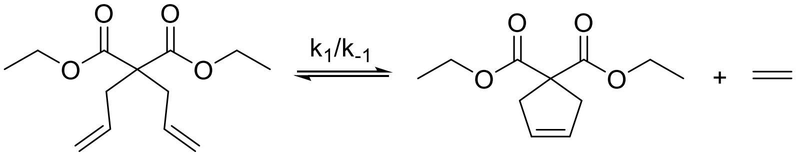

Scheme S15. Ring closing metathesis.[37] Catalysed by a Grubbs II-type catalyst.

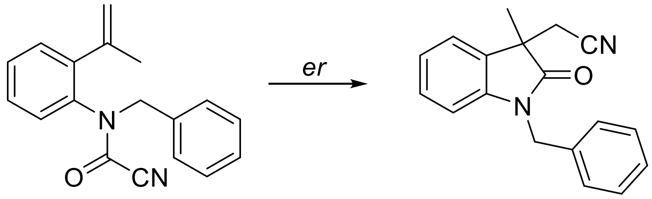

Scheme S16. Cyanoamidation.[38] Product enantioselectivity is a function of solvent polarity.

Failed approaches for KAT parameter estimations.

Other methods of KAT parameter prediction, all using COSMO-RS theory, were attempted but without success. In order to prevent other practitioners from expending their time on these unworkable models, they are briefly described here.

The obvious manner to obtain a prediction of the KAT parameters is to model the solvent effect on the ground and excited states of the dyes used in experiment. We attempted to model the difference in chemical potential between the ground state and excited state of nitroanilines in solution. Unfortunately it was difficult to obtain a reliable molecular geometry and charge distribution of an excited state without resorting to more expensive computational models. Pursuing this would have been contrary to the purpose of designing a model that is rapid to implement for novel solvents.

The second failed attempt was based on the σ-profile of the solvents. A portion of this molecular surface charge histogram served for the calculation of α (as documented earlier), but unexpectedly did not permit a reliable estimation of β. This may be due to hydrogen bond donating ability (as measured on the α scale) resulting from a single acidic proton on a molecule, whereas electron rich areas of a molecule may be delocalised and although cumulatively the σ-profile gives the appearance of a basic molecule, there may not be a focused point of charge that will engage in hydrogen bonding. Similarly, it would appear the failure to represent dipolarity (π*) is due to the σ-profile not accounting for the relative position of charges, which for a dipolar molecule are orientated to create a dipole.

The alternative σ-potential was also investigated. Each σ-potential consists of 61 datapoints. These were redefined using principal component analysis (PCA) in Origin following the procedure of Aubry.[6] Four PCA variables accounted for 97% of data variance in Marcus' KAT parameter dataset. These 4 variables did not result in accurate correlations with the KAT parameters, and were especially poor for π*. The σ-potential is based on interactions with point charges, which could in theory approximate hydrogen bonding, but not dipole-dipole interactions between molecules.

The final method was to use the σ-moments generated as part of the description of molecules in COSMO-RS theory. These parameters have been used to successfully estimate the Abraham solute parameters.[39] It was found the statistically relevant σ-moments (by regression analysis) were not logical descriptors of the KAT parameters: for instance the two relevant independent variables that produced a prediction of β actually represent the hydrogen bond donating ability of the solvent, which is what α describes. This method was not pursued further due to logical inconsistencies such as this.

Comparing different probes.

There are a number of enolisable substrates that have equilibrium constants dictated by solvent polarity, and could be superior to methyl acetoacetate and dimedone for predicting KAT parameters in virtual experiments. ethyl **acetoacetate** and dimedone.

Table S5 demonstrates that no improvement could be made by substituting *t*-butyl picolyl ketone in for methyl acetoacetate or by using anthracene ketone in place of dimedone. The fact that each probe's solvent dataset is

different (due to the availability of experimental data) means this is not a thorough analysis, but it is indicative of the quality of the virtual ln(K_T) accuracy with methyl acetoacetate and dimedone.

Table S5. A comparison of virtual equilibria with experimental data and described as a LSER.

	COSMOtherm ln(K_T) accuracy	
Probe	Compared to experimental ln(K) data	As LSER equation with KAT parameters
Methyl acetoacetate	r = 0.965	r = 0.938
t-Butyl picolyl ketone[40]	r = 0.778	r = 0.628 (r = 0.880, aprotic solvents only)
Dimedone	r = 0.967	r = 0.973
Anthracene ketone[41]	r = 0.920	r = 0.888

Direct equilibrium calculations.

It is prudent to reiterate why it is important to calculate the KAT parameters when it is possible to directly model individual processes. The primary reason is because access to the (calculated) KAT parameters then allows us to build a catalogue of potential solvents we have designed, from which to pick suitable candidates depending on what application we investigate next. The method can be reproduced by other COSMOtherm users, while the data disclosed here for commercially available solvents can be used by all. Working on an application-by-application basis would mean intuitively (or randomly) creating or selecting potential solvents and performing the virtual reaction each time, with no predictable relevance to other applications. Another benefit is that with calculated KAT parameters, reaction kinetics can be modelled (albeit indirectly) with COSMO-RS, a thermodynamic model. The computation speed for the calculation of β or π^* (from virtual tautomerisations) is a few seconds. Computational kinetics and equilibria studies are often successful in obtaining the right order of a series of rate constants or equilibrium constants, but the absolute values are harder to obtain without referencing experimental data. The KAT parameters operate on a normalised scale that automatically corrects this systematic error without having to normalise each case study individually.

Three virtual equilibria were calculated in COSMOtherm to show this limitation. This information is in the supplementary spreadsheet. The isomerisation of 3-benzoyl camphor (case study 3),[33] 2-isopropyl-5-methoxy-1,3-dioxane (case study 4),[33] and the energy difference between the rotomers of chloroacetaldehyde (case study 5),[33] show reasonable proportionality between virtual experiments and reality. However, as is true of the tautomerisation of methyl acetoacetate and dimedone, the correlation is strong but not necessarily in absolute terms. This is resolved when calculated KAT parameters are used. When directly modelling the reaction equilibrium of more complex systems (case study 1 and 2), the results had no similarity to experiment. This is in contrast to the successful free energy relationship approach using KAT parameters.[5]

Section 6. Michael addition

2-(3-Oxo-1,3-diphenyl-propyl)-malonic acid dimethyl ester (4).

Dimethyl malonate (**3**, 1.2 mmol, 0.156 g) and *trans*-chalcone (1 mmol, 0.208 g) were dissolved in the chosen solvent (5 mL), potassium phosphate (0.17 mmol) added, and the resulting suspension stirred at room temperature for 24 hours. Samples were taken at 15, 30, 45, 60, 120 and 240 minutes, and finally at 24 hours. Analysis was performed with a Hewlett-Packard 6890 series gas chromatograph using a flame ionisation detector and a ZB-HT5 fused silica column (30 m x 0.25 mm x 0.25 μm). The inlet was set at a temperature of 300 °C with a split ratio of 60, and an initial oven temperature of 50 °C before ramping at 30 °C min^{-1} to 300 °C, which was then held for 5 minutes. The total run time for each sample was 13 minutes 20 seconds. Kinetic profiles were created using the integrated second order rate law as explained by Wells *et al* (equation 3).[27]

$$k_2t = \frac{1}{x-[\mathbf{3}]} \ln \frac{[\mathbf{3}](x-[\mathbf{4}])}{x([\mathbf{3}]-[\mathbf{4}])} \qquad (3)$$

Where: [**3**] = initial concentration of first reactant; x = initial concentration of second reactant; [**4**] = concentration of product.

Isolation of the product was only undertaken for the purpose of characterisation to verify the intended reaction had occurred. To do so, the base was removed by filtration, washed with acetone and the combined organic phase concentrated *in vacuo* to give 2-(3-oxo-1,3-diphenyl-propyl)-malonic acid dimethyl ester as a white powder. Analysis was consistent with the literature.[42]

Michael addition free energy relationship.

A multivariate regression analysis was conducted to establish the relationship between ln(k) and solvent polarity using the values in Table S6. P-values were used to discard variables that were not statistically relevant (namely α and π^*, the addition of which did not improve the correlation). The resulting empirical linear solvation energy relationship is shown as equation 4, and the values of ln(k) when solving the equation with experimental and calculated values of β and Vm are given in Table S7. 2-Methyltetrahydrofuran (2-MeTHF) is not included in the data used to construct equation 4. As an indication as to whether purely calculated KAT parameters would serve as the basis of a free energy relationship, uncorrected (equation 5) and corrected (equation 6) values of β were used alongside the calculated molar volume of solvents to replicate the free energy relationship (also without 2-MeTHF). The accuracy of these free energy relationships (judged by the Pearson correlation coefficient, r) are essentially equivalent, and the magnitude of the coefficients is similar, suggesting it is possible to use calculated KAT parameters instead of experimental values to construct free energy relationships if required.

$$\ln(k) = -5.16 + 5.59\cdot\beta - 0.039\cdot V_m \qquad (r = 0.887) \qquad (4)$$

$$\ln(k) = -4.50 + 4.77\cdot\beta - 0.041\cdot V_m \qquad (r = 0.891) \qquad (5)$$

$$\ln(k) = -4.90 + 5.72\cdot\beta - 0.041\cdot V_m \qquad (r = 0.884) \qquad (6)$$

Table S6. The experimental variables in the Michael addition.

Solvent	Experimental ln(k)	β	V_m /cm^{-3}·mol^{-1}
p-Xylene	-9.54	0.12	123.3
Eucalyptol	-8.24	0.61	167.2
Ethyl acetate	-7.42	0.45	97.8
Butyl acetate	-7.13	0.45	132.1
Propylene carbonate	-6.14	0.40	85.1
Dimethyl carbonate	-5.94	0.32	84.2
2-MeTHF	-4.91	0.58	100.2

Table S7. Calculated rate constants using experimental and predicted parameters.

Solvent	Calculated ln(k)		
	Empirical	From calculated β (uncorrected) and predicted V_m	From calculated β (corrected) and predicted V_m
p-Xylene	-9.24	-9.65	-9.23
Eucalyptol	-8.19	-7.90	-8.09
Ethyl acetate	-6.42	-6.58	-6.49
Butyl acetate	-7.74	-7.92	-7.83
Propylene carbonate	-6.21	-6.50	-6.42
Dimethyl carbonate	-6.62	-6.70	-6.62
2-MeTHF	-5.78	-5.44	-5.70
MAE (without 2-MeTHF)	0.45	0.53	0.51
MAE (with 2-MeTHF)	0.51	0.53	0.55
r (without 2-MeTHF)	0.887	0.880	0.884
r (with 2-MeTHF)	0.912	0.922	0.919

Methyl 1,2,5,6-tetrahydro-1-(4-methoxyphenyl)-4-[(4-methoxyphenyl)amino]-2,6-diphenyl-3-pyridine-3-carboxylate (5).

To a solution of *p*-anisidine (0.517 g, 4.2 mmol) in the chosen solvent (1 mL), was added indium trichloride hydrate (0.159 g, 33 mol%), benzaldehyde (0.424 g, 4.0 mmol) and methyl acetoacetate (**1**, 0.323 g, 2.0 mmol). The mixture was stirred under air at room temperature for 16 hours. The resulting precipitate was isolated by filtration, washed with methanol, and recrystallised from methanol-dichloromethane to give methyl 1,2,5,6-tetrahydro-1-(4-methoxyphenyl)-4-[(4-methoxyphenyl)amino]-2,6-diphenyl-3-pyridine-3-carboxylate (**5**, up to 73%). δH(400 MHz; $CDCl_3$) 2.64 (1 H, m, CH_2), 2.79 (1 H, m, CH_2), 3.64 (3 H, s, CH_3), 3.73 (3 H, s, CH_3), 3.89 (3 H, s, CH_3), 5.03 (1 H, m, CH), 6.16 (2 H, d, *J* 9, Ar-CH), 6.32 (1 H, s, CH), 6.42 (2 H, d, *J* 9, Ar-CH), 6.60 (2 H, d, *J* 9, Ar-CH), 6.65 (2 H, d, *J* 9, Ar-CH), 7.12-7.32 (10 H, m, Ar-CH), 10.08 (1 H, s, NH). *m/z* 521.2445 (M + H^+, calculated 521.2435, ESI). Analysis is consistent with examples in the literature.[43]

Solubility study.

The tetrahydropyridine **5** was determined to have Hansen solubility parameters of δ_D = 20.4 MPa^{½}, δ_P = 10.0 MPa^{½}, δ_H = 4.6 MPa^{½}. In theory solvents with similar Hansen solubility parameters will dissolve this substrate, and dissimilar solvents will not. The boundary between solvents and non-solvents can be plotted as the radius of a sphere centred on the solute within the three dimensions defined by the Hansen solubility parameters. The radius (Ra) can only be determined experimentally, and was found to be 9.9 MPa^{½}. For this study a soluble system was defined as a 0.5 M solution. In some cases a suspension occurred, in which case the solvent was designated as a poor solvent for the purpose of calculating the Hansen sphere. In Table S8 the test solvents for the solubility study are listed in order of increasing 'relative energy difference' (RED), defined as the vector between solute and solvent in Hansen space divided by Ra. Thus a solvent with a RED equal or less than 1 theoretically should dissolve the substrate. As an empirical theorem the definition of the Hansen sphere is not precise, and there are 4 solvents at the sphere boundary wrongly in or out of the sphere. One solvent is a clear outlier, that being 2-MeTHF. These contradictions are marked with an asterisk in Table S8. The solvents for this solubility study were chosen for their diversity in order to obtain a strong fit for the Hansen sphere.

Table S8. The solvent set used to define the Hansen sphere for the tetrahydropyridine substrate. *Outlying data.

Solvent	δ_D /MPa½	δ_P /MPa½	δ_H /MPa½	Solubility	RED
Nitrobenzene	20.0	10.6	3.1	High	0.19
N-Methyl pyrrolidone	18.0	12.3	7.2	High	0.60
Dimethyl Isosorbide	17.6	7.1	7.5	High	0.70
γ-Valerolactone	16.8	11.4	6.7	High	0.77
Propylene Carbonate	20.0	18.0	4.1	High	0.83
2-MeTHF	16.9	5.0	4.3	Suspension	0.86*
Chloroform	17.8	3.1	5.7	High	0.87
N,N-Dimethyl acetamide	16.8	11.5	9.4	High	0.89
Tetrahydrofuran	16.8	5.7	8.0	High	0.91
Guaiacol	18.0	7.0	12.0	High	0.94
Dimethyl sulphoxide	18.4	16.4	10.2	Suspension	0.96*
N,N-Dimethyl formamide	17.4	13.7	11.3	High	0.99
Tetrahydrofurfuryl alcohol	17.8	8.2	12.9	High	1.00
Solketal	16.8	7.5	11.0	Low	1.00
p-Cymene	17.3	2.4	2.4	Low	1.01
Sulfolane	17.8	17.4	8.7	High	1.01*
Acetone	15.5	10.4	7.0	High	1.02*
Toluene	18.0	1.4	2.0	High	1.02*
d-Limonene	17.2	1.8	4.3	Low	1.04
Ethyl Acetate	15.8	5.3	7.2	Suspension	1.07
Dimethyl Carbonate	15.5	8.6	9.7	Low	1.12
Ethyl Lactate	16.0	7.6	12.5	Low	1.22
Nitromethane	15.8	18.8	6.1	Suspension	1.30
Acetonitrile	15.3	18.0	6.1	Low	1.33
Diethyl Ether	14.5	2.9	4.6	Low	1.39
Triethylamine	15.5	0.4	1.0	Low	1.43
Propionic Acid	14.7	5.3	12.4	Suspension	1.47
Acetic Acid	14.5	8.0	13.5	Low	1.50
1-Butanol	16.0	5.7	15.8	Low	1.50
Heptane	15.3	0.0	0.0	Low	1.51
t-Butanol	15.2	5.1	14.7	Suspension	1.54
PEG-400	16.0	12.5	18.6	Suspension	1.69
Triethylene glycol	16.0	12.5	18.6	Low	1.69
Ethanol	15.8	8.8	19.4	Low	1.76
Propylene glycol	16.8	10.4	21.3	Low	1.84
Lactic Acid	17.3	10.1	23.3	Low	1.99
Methanol	14.7	12.3	22.3	Low	2.14
Ethylene Glycol	17.0	11.0	26.0	Suspension	2.27
Glycerol	17.4	11.3	27.2	Low	2.36
Water	15.5	16.0	42.3	Low	3.98

Tetrahydropyridine synthesis free energy relationship.

Following the same principle as for the previous Michael addition case study, a free energy relationship was constructed to correlate reaction productivity with the KAT parameters. A free energy relationship was arrived at in the usual manner using the data in Table S9 (equation 7, not using data for levoglucosanol), which can then be used to predict yields (Table S10). The calculated π* value of levoglucosanol was obtained from the virtual tautomerisation of methyl acetoacetate (ln(K) = 8.29, normalised to 0.16). The free energy relationship in equation 7 was replicated using uncorrected (equation 8) and corrected (equation 9) values of π* to form the correlation instead of experimental values of π* (also without levoglucosanol). The proportionality between ln(K′) and π* is retained.

$$\ln(K') = -0.44 + 1.44 \cdot \pi^* \qquad (r = 0.860) \qquad (7)$$

$$\ln(K') = -0.17 + 0.87 \cdot \pi^* \qquad (r = 0.647) \qquad (8)$$

$$\ln(K') = -0.31 + 1.20 \cdot \pi^* \qquad (r = 0.858) \qquad (9)$$

Table S9. The experimental variables in the multi-component tetrahydropyridine synthesis.

Solvent	Yield	Experimental ln(K′)	π*
Diethyl ether	45%	-0.22	0.27
2-MeTHF	55%	0.21	0.53
t-Butanol	61%	0.43	0.41
Methanol	62%	0.51	0.60
Acetonitrile (MeCN)	63%	0.54	0.75
N-Butyl pyrrolidone	67%	0.69	0.77
Levoglucosanol	73%	0.97	0.89
Dihydrolevoglucosenone	0% (multiple products)		0.93
Dimethyl sulphoxide	0% (imine isolated)		1.00
Glycerol	8% (multiple products)		1.04

Table S10. Calculated yields using experimental and predicted parameters.

Solvent	Calculated yield (ln(K′) in brackets)		
	Empirical	From calculated π* (uncorrected)	From calculated π* (corrected)
Diethyl ether	49% (-0.05)	51% (0.05)	48% (-0.08)
2-MeTHF	58% (0.32)	55% (0.19)	57% (0.27)
t-Butanol	54% (0.15)	58% (0.30)	54% (0.16)
Methanol	60% (0.42)	67% (0.71)	59% (0.35)
Acetonitrile	65% (0.64)	73% (0.98)	65% (0.61)
N-Butyl pyrrolidone	66% (0.67)	59% (0.37)	70% (0.87)
Levoglucosanol	70% (0.84)	68% (0.76)	71% (0.90)
MAE (ln(K′), without levoglucosanol)	0.11	0.19	0.12
MAE (ln(K′), with levoglucosanol)	0.11	0.20	0.12
r (ln(K′), without levoglucosanol)	0.860	0.647	0.858
r (ln(K′), with levoglucosanol)	0.909	0.699	0.905

6,8-Dioxabicyclo[3.2.1]octan-4-ol (levoglucosanol).

To a solution of sodium borohydride (3.63 g, 96 mmol) in water (40 mL) in an ice bath was added dihydrolevoglucosenone (7.57 g, 60 mmol) dropwise. The mixture was stirred overnight, and allowed to warm to the ambient temperature. Then chloroform (3 x 50 mL) was added and separated, and the combined organic extracts combined, dried with magnesium sulphate, and concentrated *in vacuo* to give 6,8-dioxabicyclo[3.2.1]octan-4-ol as a colourless liquid (84%). δH(400 mHz; $CDCl_3$) 1.52 (2 H, m, CH_2), 1.85 (1 H, m, CH_2), 1.98 (1 H, m, CH_2), 2.26 (1 H, d, *J* 9, OH), 2.54 (1 H *of minor diastereoisomer*, d, *J* 9, OH), 3.54 (1 H, m, CH_2), 3.80 (2 H, m, CH & CH_2), 3.88 (1 H *of minor diastereoisomer*, d, *J* 7, CH), 4.45 (1 H, s, CH), 5.27 (1 H, s, CH). *m/z* 153.0522 (M + Na^+, calculated 153.0522, ESI). Analysis is consistent with the literature.[44] The product consists of diastereoisomers in an approximate 6:1 molar ratio favouring the *endo*-diastereomer (Figure S11).

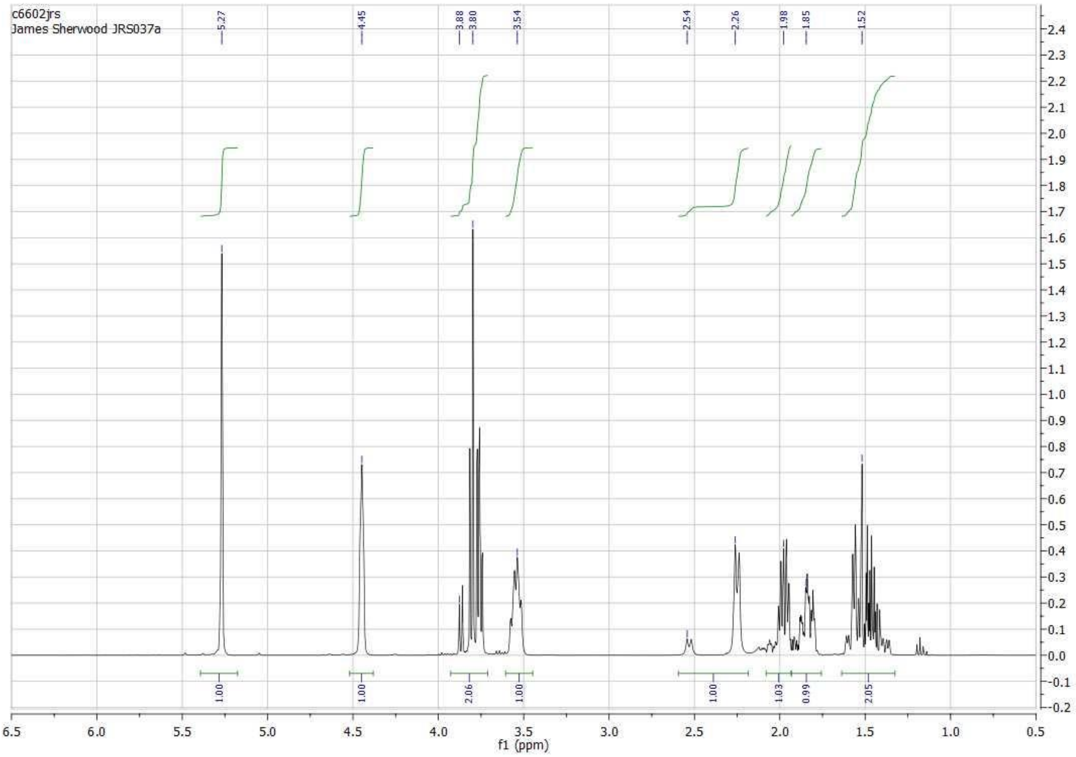

Figure S11. ^{1}H-NMR spectrum of levoglucosanol. Resolved diasteroisomeric signals are integrated together.

Supplementary references

[1] Marcus, Y. The properties of organic liquids that are relevant to their use as solvating solvents. *Chem. Soc. Rev.* **1993**, *22*, 409-416.

[2] Taft, R.W.; Kamlet, M.J. The solvatochromic comparison method. 2. the α-scale of solvent hydrogen-bond donor (HBD) acidities. *J. Am. Chem. Soc.* **1976**, *98*, 2886-2894.

[3] Marcus, Y. The effectiveness of solvents as hydrogen bond donors. *J. Solution Chem.* **1991**, *20*, 929-944.

[4] Crowhurst, L.; Mawdsley, P.R.; Perez-Arlandis, J.M.; Salter, P.A.; Welton, T. Solvent-solute interactions in ionic liquids. *Phys. Chem. Chem. Phys.* **2003**, *5*, 2790-2794.

[5] Clark, J.H.; Macquarrie, D.J.; Sherwood, J. The combined role of catalysis and solvent effects on the Biginelli reaction: improving efficiency and sustainability. *Chem. Eur. J.* **2013**, *19*, 5174-5182.

[6] Durand, M.; Molinier, V.; Kunz, W.; Aubry, J.-M. Classification of organic solvents revisited by using the COSMO-RS approach. *Chem Eur. J.* **2011**, *17*, 5155-5164.

[7] Eckert, F.; Klamt, A. Fast solvent screening via quantum chemistry: COSMO-RS approach. *AIChE Journal.* **2002**, *48*, 369-385.

[8] Klamt, A.; Jonas, V.; Bürger, T.; Lohrenz, J.C. Refinement and parametrization of COSMO-RS. *J. Phys. Chem. A.* **1998**, *102*, 5074-5085.

[9] Klamt, A. Conductor-like screening model for real solvents: a new approach to the quantitative calculation of solvation phenomena. *J. Phys. Chem.* **1995**, *99*, 2224-2235.

[10] Klamt, A. The COSMO and COSMO-RS solvation models. *WIREs Comput. Mol. Sci.* **2018**, *8*, e1338.

[11] Diorazio, L.J.; Hose, D.R.J.; Adlington, N.K. Toward a more holistic framework for solvent selection. *Org. Process Res. Dev.* **2016**, *20*, 760-773.

[12] Waghorne, W.E.; O'Farrell, C. Solvent basicity, a study of Kamlet-Taft β and Gutmann DN values using computationally derived molecular properties. *J. Solution Chem.* **2018**, *47*, 1609-1625.

[13] Palomar, J.; Torrecilla, J.S.; Lemus, J.; Ferro, V.R.; Rodríguez, F. A COSMO-RS based guide to analyze/quantify the polarity of ionic liquids and their mixtures with organic cosolvents. *Phys. Chem. Chem. Phys.* **2010**, *12*, 1991-2000.

[14] Reichardt, C. Solvatochromic dyes as solvent polarity indicators. *Chem. Rev.* **1994**, *94*, 2319-2358.

[15] Jessop, P.G.; Jessop, D.A.; Fu, D.; Phan, L. Solvatochromic parameters for solvents of interest in green chemistry. *Green Chem.* **2012**, *14*, 1245-1259.

[16] Mouret, A.; Leclercq, L.; Mühlbauer, A.; Nardello-Rataj, V. Eco-friendly solvents and amphiphilic catalytic polyoxometalate nanoparticles: a winning combination for olefin epoxidation. *Green Chem.* **2014**, *16*, 269-278.

[17] Parker, H.L.; Sherwood, J.; Hunt, A.J.; Clark, J.H. Cyclic carbonates as green alternative solvents for the Heck reaction. *ACS Sustainable Chem. Eng.* **2014**, *2*, 1739-1742.

[18] Sherwood, J. Bio-Based Solvents in Organic Synthesis, PhD, University of York, York, 2013.

[19] Clark, J.H.; Macquarrie, D.J.; Sherwood, J. A quantitative comparison between conventional and bio-derived solvents from citrus waste in esterification and amidation kinetic studies. *Green Chem.* **2012**, *14*, 90-93.

[20] Sherwood, J.; De bruyn, M.; Constantinou, A.; Moity, L.; McElroy, C.R.; Farmer, T.J.; Duncan, T.; Raverty, W.; Hunt, A.J.; Clark, J.H. Dihydrolevoglucosenone (Cyrene) as a bio-based alternative for dipolar aprotic solvents. *Chem. Commun.* **2014**, *50*, 9650-9652.

[21] Alves Costa Pacheco, A.; Sherwood, J.; Zhenova, A.; McElroy, C.R.; Hunt, A.J.; Parker, H.L.; Farmer, T.J.; Constantinou, A.; De bruyn, M.; Whitwood, A.C.; Ravery, W.; Clark, J.H. Intelligent approach to solvent substitution: the identification of a new class of levoglucosenone derivatives. *ChemSusChem* **2016**, *9*, 3503-3512.

[22] Byrne, F.; Forier, B.; Bossaert, G.; Hoebers, C.; Farmer, T.J.; Clark, J.H.; Hunt, A.J. 2,2,5,5-Tetramethyltetrahydrofuran (TMTHF): a non-polar, non-peroxide forming ether replacement for hazardous hydrocarbon solvents. *Green Chem.* **2017**, *19*, 3671-3678.

[23] Sherwood, J.; Parker, H.L.; Moonen, K.; Farmer, T.J.; Hunt, A.J. N-Butylpyrrolidinone as a dipolar aprotic solvent for organic synthesis. *Green Chem.* **2016**, *18*, 3990-3996.

[24] Byrne, F.; Forier, B.; Bossaert, G.; Hoebers, C.; Farmer, T.J.; Hunt, A.J. A methodical selection process for the development of ketones and esters as bio-based replacements for traditional hydrocarbon solvents. *Green Chem.* **2018**, *20*, 4003-4011.

[25] Lagalante, A.F.; Wood, C.; Clarke, A.M.; Bruno, T.J. Kamlet–Taft solvatochromic parameters for 25 glycol ether solvents and glycol ether aqueous solutions. *J. Solution Chem.* **1998**, *27*, 887-900.

[26] Skrzypczak, A.; Neta, P. Rate constants for reaction of 1,2-dimethylimidazole with benzyl bromide in ionic liquids and organic solvents. *Int. J. Chem. Kinet.* **2004**, *36*, 253-258.
[27] Wells, T.P.; Hallett, J.P.; Williams, C.K.; Welton, T. Esterification in ionic liquids: the influence of solvent basicity. *J. Org. Chem.* **2008**, *73*, 5585-5588.
[28] Ab Rani, M.A.; Brant, A.; Crowhurst, L.; Dolan, A.; Lui, M.; Hassan, N.H.; Hallett, J.P.; Hunt, P.A.; Niedermeyer, H.; Perez-Arlandis, J.M.; Schrems, M.; Welton, T.; Wilding, R. Understanding the polarity of ionic liquids. *Phys. Chem. Chem. Phys.* **2011**, *13*, 16831-16840.
[29] Bini, R.; Chiappe, C.; Llopsis Mestre, V.; Pomelli, C.S.; Welton, T. A rationalization of the solvent effect on the Diels–Alder reaction in ionic liquids using multiparameter linear solvation energy relationships. *Org. Biomol. Chem.* **2008**, *6*, 2522-2529.
[30] Teles, A.R.R.; Capela, E.V.; Carmo, R.S.; Coutinho, J.A.P.; Silvestre, A.J.D.; Freire, M.G.. Solvatochromic parameters of deep eutectic solvents formed by ammonium-based salts and carboxylic acids. *Fluid Phase Equilibria* **2017**, *448*, 15-21.
[31] Dietz, C.H.J.T.; Kroon, M.C.; van sint Annaland, M.; Gallucci, F. Thermophysical properties and solubility of different sugar-derived molecules in deep eutectic solvents. *J. Chem. Eng. Data* **2017**, *62*, 3633-3641.
[32] Abbott, A.P.; Harris, R.C.; Ryder, K.S.; D'Agostino, C.; Gladden, L.F.; Mantle, M.D. Glycerol eutectics as sustainable solvent systems. *Green Chem.* **2011**, *13*, 82-90.
[33] Reichardt, C.; Welton, T. *Solvents and Solvent Effects in Organic Chemistry*, 4th ed.; Wiley-VCH, Weinheim, Germany, 2011.
[34] Abraham, M.H. Solvent effects on the free energies of the reactants and transition states in the Menschutkin reaction of trimethylamine with alkyl halides. *J. Chem. Soc. D.* **1969**, 1307-1308.
[35] Abraham, M.H.; Doherty, R.M.; Kamlet, M.J.; Harris, J.M.; Taft, R.W. Linear solvation energy relationships. part 37. an analysis of contributions of dipolarity–polarisability, nucleophilic assistance, electrophilic assistance, and cavity terms to solvent effects on t-butyl halide solvolysis rates. *J. Chem. Soc. Perkin Trans. 2* **1987**, 913-920.
[36] Iemhoff, A.; Sherwood, J.; McElroy, C.R.; Hunt, A.J. Towards sustainable kinetic resolution, a combination of bio-catalysis, flow chemistry and bio-based solvents. *Green Chem.* **2018**, *20*, 136-140.
[37] Adjiman, C.S.; Clarke, A.J.; Cooper, G.; Taylor, P.C. Solvents for ring-closing metathesis reactions. *Chem. Commun.* **2008**, 2806-2808.
[38] Frost, G.B.; Serratore, N.A.; Ogilvie, J.M.; Douglas, C.J. Mechanistic model for enantioselective intramolecular alkene cyanoamidation via palladium-catalyzed C-CN bond activation. *J. Org. Chem.* **2017**, *82*, 3721-3726.
[39] Zissimos, A.M.; Abraham, M.H.; Klamt, A.; Eckert, F.; Wood, J. A comparison between the two general sets of linear free energy descriptors of Abraham and Klamt. *J. Chem. Inf. Comput. Sci.* **2002**, *42*, 1320-1331.
[40] Roussel, R.; Oteyza de Guerrero, M.; Spegt, P.; Galin, J.C. Solvent effects on the tautomerisation of t-butyl 2-picolyl and t-butyl quinaldyl ketones. *J. Heterocyclic Chem.* **1982**, *19*, 785-796.
[41] Mills, S.G.; Beak, P. Solvent effects on keto-enol equilibria: tests of quantitative models. *J. Org. Chem.* **1985**, *50*, 1216-1224.
[42] Agostinho, M.; Kobayashi, S. Strontium-catalyzed highly enantioselective Michael additions of malonates to enones. *J. Am. Chem. Soc.* **2008**, *130*, 2430-2431.
[43] Clarke, P.A.; Zaytsev, A.V.; Whitwood, A.C. Pot, atom, and step economic (PASE) synthesis of highly substituted piperidines: a five-component condensation. *Synthesis* **2008**, 3530-3532.
[44] Kadota, K.; Kurusu, T.; Taniguchi, T.; Ogasawara, K. Lipase-mediated synthesis of both enantiomers of levoglucosenone from acrolein dimer. *Adv. Synth. Catal.* **2011**, *343*, 618-623.
